# Template-directed ligation on repetitive DNA sequences: a chemical method to probe the length of Huntington DNA†
†Electronic supplementary information (ESI) available. See DOI: 10.1039/c4sc01974a



**DOI:** 10.1039/c4sc01974a

**Published:** 2014-09-16

**Authors:** Anika Kern, Oliver Seitz

**Affiliations:** a Institut für Chemie , Humboldt-Universität zu Berlin , Brook-Taylor-Straße 2 , 12489 Berlin , Germany . Email: oliver.seitz@chemie.hu-berlin.de ; Fax: +49-30-2093-7266

## Abstract

Several genomic disorders are caused by an excessive number of DNA triplet repeats. We developed a DNA-templated reaction in which product formation occurs only when the number of repeats exceeds a threshold indicative for the outbreak of Chorea Huntington. The combined use of native chemical PNA ligation and auxiliary DNA probes enabled reactions on templates obtained from human genomic DNA.

## Abstract 2

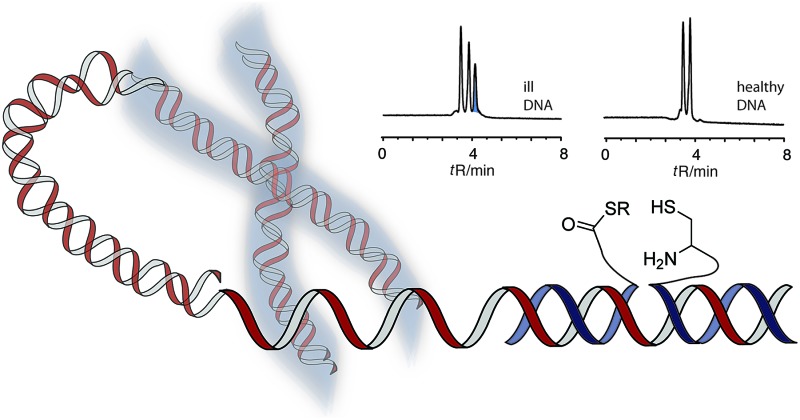
Several genomic disorders are caused by an excessive number of DNA triplet repeats.

## 

Nucleic acid controlled chemical reactions are emerging as useful tools for nucleic acid diagnosis and imaging.^1–6^ Reactive probes offer advantages to the commonly used non-reactive hybridization probes.^7^ For example, DNA/RNA-controlled reactions proceed with very high sequence specificity – at the single nucleotide level if desired – because adjacent and simultaneous annealing of two probe molecules is required to trigger the chemical reaction. Furthermore, chemical reactions can occur with turnover in template, which offers opportunities for the amplified detection of product signals.^7,8^ While significant efforts have been invested into the development of new chemistries applied in challenging environments such as in cell lysates, during the polymerase chain reaction and within living cells, comparatively little attention has been paid to the nature of the targeted nucleic acid template itself.^9–18^ A number of genes contain repeat sequences. Several disorders such as Huntington disease, myotonic dystrophy and spinocerebellar ataxia are caused by an excessive number of trinucleotide repeats.^19–23^ For example, the expansion of triplet repeats in the human IT15 gene leads to Chorea Huntington Disease (HD).^24,25^ Alleles with less than 26 repeats are considered normal and are stable, but alleles that contain 27–36 triplet repeats may expand during meiosis, hence leading to increased repeats in the next generation. Eventually, alleles with more than 36 triplet repeats lead to HD pathology.^26,27^

We envisioned that a nucleic acid directed reaction could be designed to provide product required that the number of repeat sequences exceeds a critical threshold value. This idea was based on the notion that the number of triplet repeats in the template should correlate with the effective concentration of template available for binding of the reactive probes.

Our design of a reaction system that discriminates between repeat sequence DNA of varied length involves the native chemical ligation of peptide nucleic acid (PNA)-based probes (Scheme 1).^28–32^ PNA is a DNA analogue that has high affinity for complementary DNA.^33–35^ This facilitates hybridization and reactions on folded nucleic acid targets (such as long DNA repeat sequences). The method requires two reactive PNA probes, one of which is equipped with a C-terminal thioester moiety (**I**) while the other bears an N-terminal cysteine residue (**II**). Hybridization with the complementary DNA sequence brings the reactive groups into proximity and triggers the native chemical ligation, whereas no reaction should occur in the absence of template.^30^

**Scheme 1:**
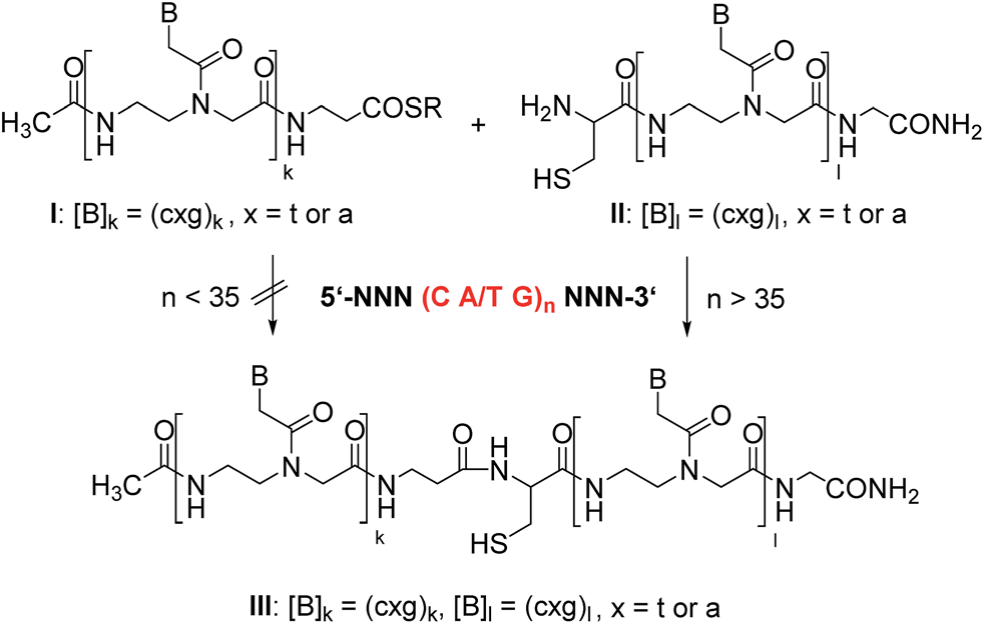
Principle of a native chemical ligation of PNA probes (**I** and **II**), in which formation of product **III** is indicative for a critical length of a triplet repeat DNA template.

In the initial phase of probe development, we compared a probe set directed against the CAG repeats in the Huntington coding strand with a probe set targeted against the CTG repeats of the corresponding non-coding strand. The latter was favoured by us owing to difficulties in the synthesis of (CTG)_*n*_ containing PNA probes. Next we investigated whether templated reactions occur on repeat sequence templates. The CAG-containing PNA thioester **1** and cysteinyl-PNA **2** were allowed to react in the absence and presence of a size-matched synthetic CTG-repeat DNA template (Fig. 1a). The formation of product **3** was monitored by reversed phase-ultra high performance chromatography (RP-UPLC, Fig. 1b). This enabled the analysis of picomol amounts within short time (4 minutes). During the optimization of the native chemical ligation between **1** and **2** we noticed a surprisingly high background reaction in absence of template (Fig. S37†). To avoid probe–probe interactions the reaction temperature was increased to 50 °C. We next added 200 nM CTG-repeat DNA templates of varied length. This concentration is within the range of amplicons obtained after PCR. Of note, the rates of native chemical PNA ligation proved critically dependent on the number of repeats (Fig. 1c). The positive correlation between product yield and repeat length extended until 20 CTG repeats (Fig. 1d). Then, at 24 CTG repeats the efficiency of the PNA ligation suddenly dropped. Increases of probe concentration to 5 μM did not lead to an improvement. Melt experiments (Table S1†) suggested that the template adopted a superstructure that had significantly higher stability ((CTG)_20_, *T*_M_ = 43 °C; (CTG)_32_, *T*_M_ = 66 °C) than the complexes formed between the reactive PNA probes and the template (**1**·(CTG)_4_, *T*_M_ = 39 °C; **2**·(CTG)_4_, *T*_M_ = 38 °C). The PNA probes were elongated by three nucleotides to increase the template affinity. However, the rather large extent of background ligation was problematic (Fig. S38†).

**Fig. 1:**
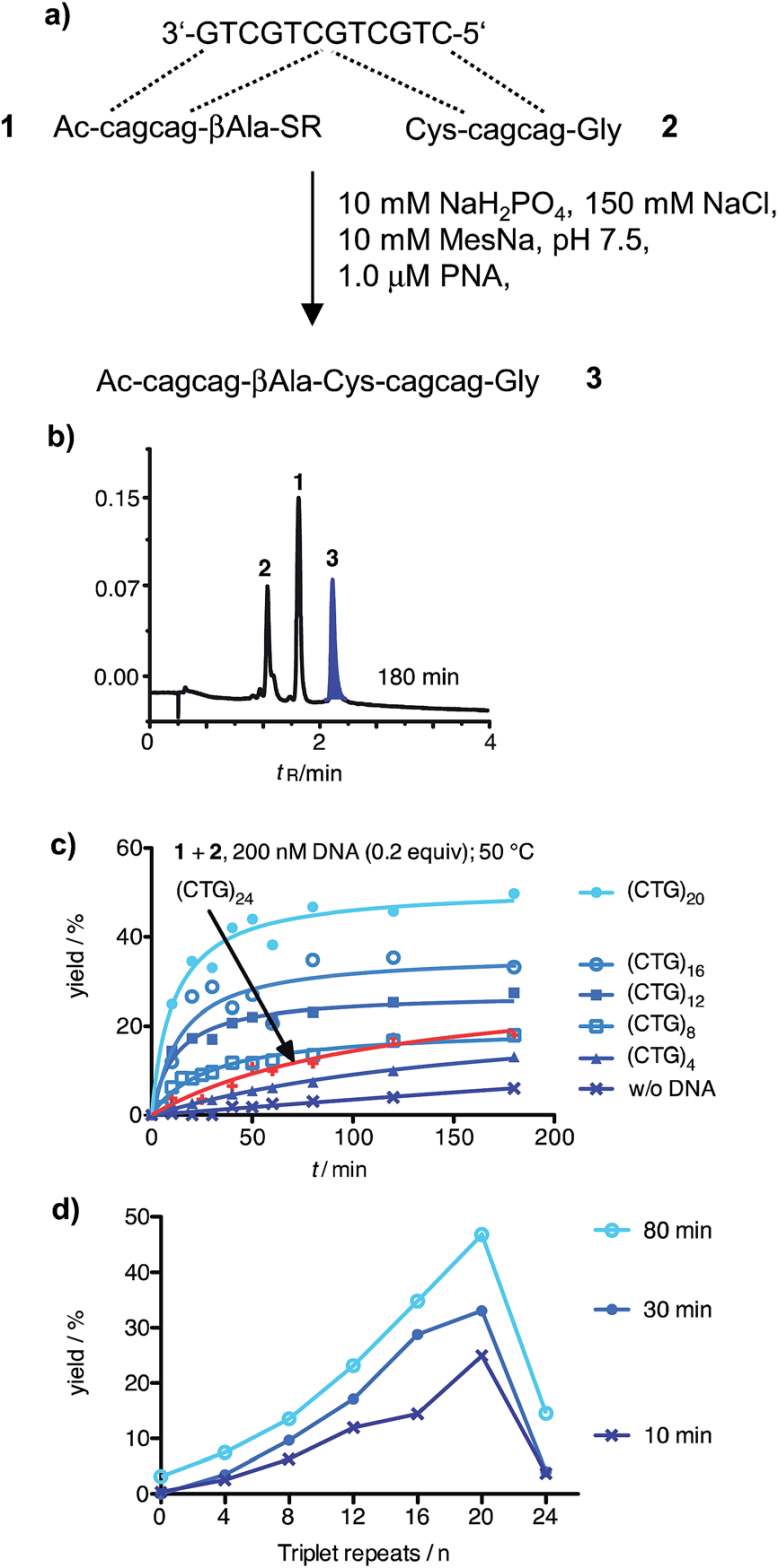
(a) DNA templated native chemical PNA ligation between **1** and **2** at equimolar probe/template ratio; (b) UPLC analysis of aliquots withdrawn from the reaction mixture after the specified reaction times; (c) time course of reactions in presence of synthetic ssDNA varying in triplet repeat length; (d) fractional increase in product yield after 10 min, 30 min or 80 min. Conditions: 1 μM reactive PNA probes in buffer (10 mM NaH_2_PO_4_, 150 mM NaCl, 10 mM MesNa, pH 7.5), 200 nM template when added, 50 °C. R = (CH_2_)_2_SO_3_H.

We assumed that the use of aminomodified PNA should offer a solution to the problem of PNA–PNA interaction and, at the same time, confer the required increase of the template affinity. According to this, charge repulsion would help reduce PNA–PNA interactions whereas charge attraction would foster the formation of PNA–DNA template complexes. For that purpose, the aminoethyllysine cytidine building block c* was prepared (ESI†) and incorporated into the CAG-containing PNA thioester probe **4** (Fig. 2a). The cysteinyl-PNA **5** was equipped with a C-terminal lysine. The extent of background reaction between the aminomodified reactive probes **4** and **5** in absence of template was very small (Fig. S37†) and the scope of templated reactions was extended to sequences longer than 20 repeats. The yield of the templated ligation between **4** and **5** continued to increase until a length of 32 triplet repeats (Fig. 2b and S39†). This corresponds to the length, which may lead to the Huntington phenotype in offspring. However, the ligation efficiency was severely affected, again, when the DNA length exceeded 32 repeats (Fig. 2c). The addition of denaturating agents such as DMSO, glycerol, betaine or formamide allowed increases of product formation (Fig. S41 and S42†). But still, the fractional increase in ligation rate did not surpass a length threshold of 32 repeats.

**Fig. 2:**
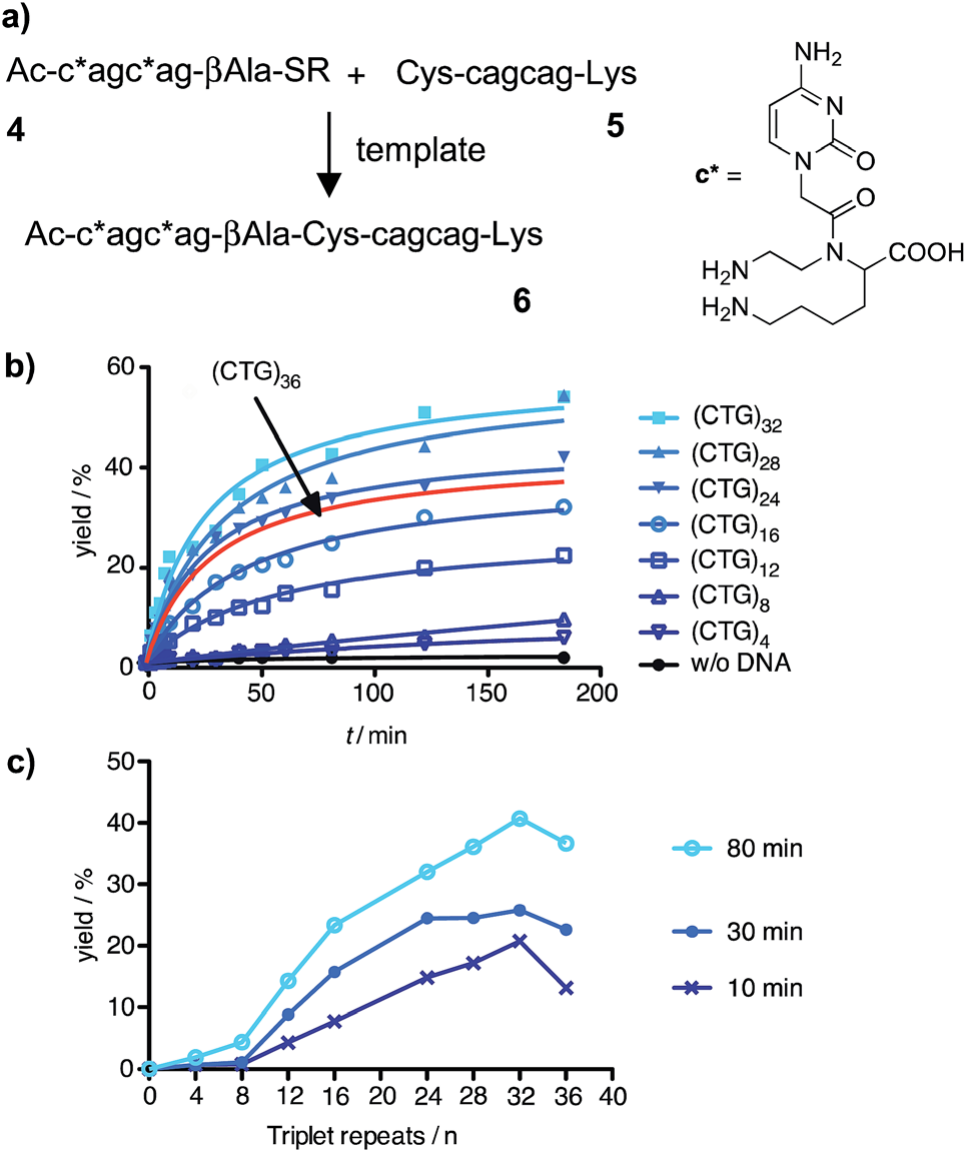
(a) Native chemical ligation reaction between aminomodified PNA probes **4** and **5**; (b) time course of product **6** formed in absence or presence of 100 nM DNA templates varying in the number of triplet repeats; (c) template dependence of yields obtained after 10 min, 30 min or 80 min. (Conditions: see Fig. 1, R = (CH_2_)_2_SO_3_H).

In previous ligation chemistries, auxiliary strands have been added to prevent reannealing of a DNA template strand produced by PCR.^11,30,36^ However, intramolecular hybridization is a very efficient process and the length requirements for oligonucleotides that block the formation of superstructures in the Huntington template were unknown. To avoid slippage along the template, we designed a blocker strand that is equipped with a 5′-terminal hexanucleotide sequence which anchored the CAG repeat sequence to the 3′-flanking region of the CTG repeat tract. The so-called partial blocker DNA strands **7** and **8** offered 12 or 28 CAG repeats for binding interactions with the CTG repeat part of the Huntington non-coding strand (Fig. 3a). The partial blockers **7** or **8** were added to the **4** + **5** ligation. Of note, the ligations proceeded remarkably well on DNA templates containing 44 triplet repeats (Fig. 3b). No product was formed when the template strand contained less triplet repeats than the partial blocker. Reactions performed in presence of the short blocker **7** provided higher ligation yields than reactions in presence of the long blocker **8**. This can be attributed to the increased number of template bases available for annealing of reactive probes. These results showed that a DNA-directed chemical reaction can distinguish between healthy and pathological DNA length.

**Fig. 3:**
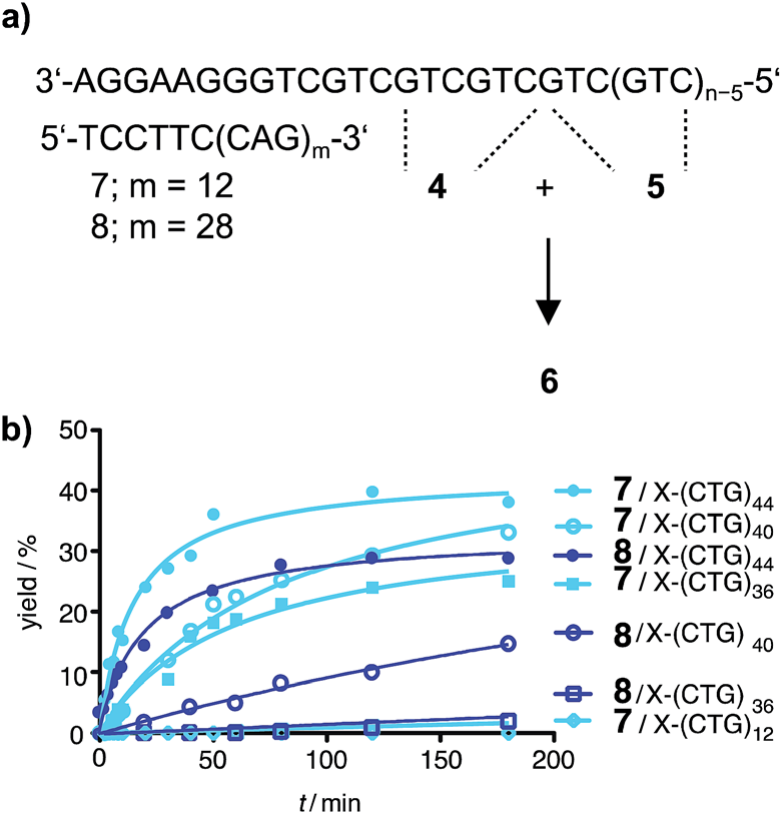
(a) Principle of native chemical PNA ligation aided by partial DNA blockers **7** and **8** and (b) time course of the product formation triggered by DNA templates varying in triplet repeats length for reaction systems involving **7** or **8**. Conditions: 1 μM reactive PNA probes in buffer (10 mM NaH_2_PO_4_, 150 mM NaCl, 10 mM MesNa, pH 7.5), 100 nM DNA template, 100 nM partial blocker, R = (CH_2_)_2_SO_3_H.

To test the reaction system under realistic conditions we analyzed human genomic DNA templates. Samples from a Chorea Huntington patient (purchased from the Coriell Institute) and human wild-type DNA were subjected to PCR, which was used to amplify the trinucleotide repeat segment of the IT15 gene. This was achieved by using a biotin-modified forward primer and an Alexa700-labeled reverse primer and adapting a known PCR method.^37,38^ The amplified DNA was captured by streptavidin-magnetic beads and NaOH treatment was used to release single stranded DNA templates (ESI†). The yield of PCR and the concentration of template strands were determined *via* fluorescence measurements. The reactive probes and partial blocker **7** were added to the PCR-DNA single strand. Aliquots were withdrawn after 0–180 min reaction time and analyzed *via* UPLC (Fig. 4). The DNA template obtained through amplification of Huntington Disease genomic DNA (HD-DNA) triggered the formation of ligation product **6** (Fig. 4b). The ligation product was detectable after 10 min reaction time (10% yield) and reached 32% in 180 minutes. By contrast, no product was formed when the reaction system involved the wild-type DNA template (Fig. 4c). The analysis was repeated with partial blocker **8** (Fig. S44 and S45†). Again, no product was formed when the template was produced from wild-type genomic DNA, whereas UPLC analysis revealed the time-dependent synthesis of ligation product **6** in reactions including the HD-DNA template. We wish to note that the signal detected *via* RP-UPLC is robust and can be obtained nearly as rapidly as by a fluorometric read-out. Based on peak height the signal-to-noise on a genomic DNA template is 46 (after 10 min reaction time).

**Fig. 4:**
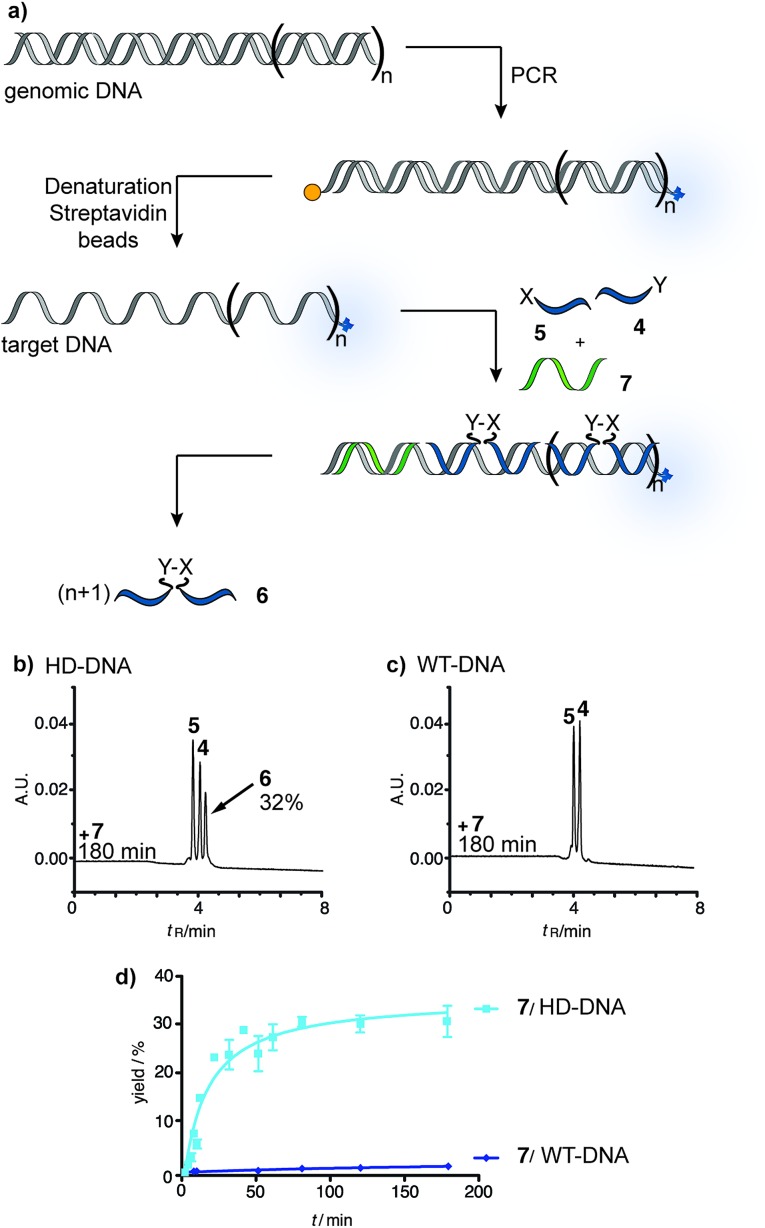
(a) Principle of native chemical PNA ligation on templates obtained *via* PCR from human genomic DNA. UPLC traces (b and c) and time course (d) of reactions performed on DNA from (b) a Chorea Huntington patient (HD-DNA) or (c) a healthy individual (WT-DNA). Conditions: see Fig. 3.

The data shows that the PNA-based reaction system responds to the length of repeat sequences in a way that enables ligation when a certain threshold is surpassed. The combined use of reactive probes and auxiliary probes provided a clear yes/no response to the diagnosis problem. The comparison with data obtained with synthetic DNA templates suggested that the HD-DNA has more than 28 repeats. The rate profile resembles reactions with >40 repeat sequences (Fig. S44 and S45†).

The reported work represents the first attempt to assess the length of triplet repeat DNA by means of a DNA-templated reaction. The PNA native chemical ligation provided detectable product after 10 min reaction time and the UPLC analysis was obtained within 4 minutes. Therefore, the time required for analysis by PNA ligation is shorter than the time required for the commonly used analysis of PCR products by gel electrophoresis. However, the need for single stranded DNA template adds capture and denaturation steps to the PCR protocol and, thereby, increases the total analysis time. Sequencing methods could, in principle, provide a more direct means of counting the number of triplet repeats. Yet, long read lengths are required in order to avoid ambiguities in alignment. The resulting need for the separation of the differently sized DNA amplicons calls for electrophoretic methods, which also are time consuming. A reduction of the analysis time required in our method should be feasible. We propose the use of PNA-based invaders such as the G-clamp modified γPNA^39^ introduced by Ly as this would make the capture/denaturation step redundant.

## Conclusions

We identified a reaction system, which allowed the discrimination between Chorea Huntington DNA and wild-type DNA. The reaction system relied on reactive PNA probes, which contained thioester and cysteinyl units required to drive a native chemical ligation reaction. Of note, aggregation of repeat sequence PNA and the formation of template superstructures presented a formidable challenge. A solution to the problem of false positive ligation and folding of large repeat templates was provided by the combined use of amino-modified PNA probes and auxiliary, non-reactive DNA hybridization probes. Under these conditions, the native chemical PNA ligation proceeded at high rate and afforded detectable signals after 10 min reaction time provided that the template was longer than the auxiliary probe (partial blocker). The length-reactivity relationship is within a range that allows the characterization of various polyglutamine diseases.
