# Supplementary material for: Template-directed ligation on repetitive DNA sequences: a chemical method to probe the length of Huntington DNA
Source: Chem Sci. 2014 Sep 16;6(1):724–8. doi: 10.1039/c4sc01974a (PMC5494559; doi:10.1039/c4sc01974a)
Supplement: Supplementary file 1 [file SC-006-C4SC01974A-s001.pdf]

Electronic Supplementary Material (ESI) for Chemical Science.
This journal is © The Royal Society of Chemistry 2014

Template-directed ligation on repetitive DNA sequences: a chemical method to probe the length of Huntington DNA

Anika Kern and Oliver Seitz*

Institut für Chemie, Humboldt-Universität zu Berlin, Brook-Taylor-Straße 2,D-12489 Berlin, Germany.

Materials and general procedures S2

Synthesis and characterization data of the aminoethyllysine cytidine building block (c*) S4

Synthesis of the PNA conjugates S22

Measurements with PNA probes S26

Melting Temperature (T_M) measurements S26

Kinetic measurements of templated reaction S27

Polymerase Chain Reaction S29

PCR conditions S30

Purification of amplified dsDNA S30

Streptavidin binding procedure S30

Abbreviation S31

References S32

Materials and general procedures

Materials: MBHA-resin was purchased from *NovaBiochem* (Schwalbach, Germany). PNA monomers were purchased from *Applied Biosystems* (Darmstadt, Germany), *ASM* (Burgwedel, Germany) and *Panagene* (Daejeon, Korea). Protected standard amino acids (Fmoc-Gly-OH, Boc-Lys(Fmoc)-OH, Boc-βAla-OH, Boc-Cys(Trt)-OH) were purchased from *ASM*, *NeoMPS* (Strasbourg, France) and *Panagene*. PyBOP was used as coupling reagent and purchased from *Chemcube* (Bochum, Germany). DNA and the biotin-containing primer was purchased HPLC purified from *BioTeZ* Berlin-Buch GmbH. Alexa700-containing primer was

purchased HPLC purified from *IBA-lifesciences GmbH* (Göttingen, Germany). Genomic Huntington DNA was purchased from the *Coriell Cell Repositories* and genomic wild-type DNA was purchased from *Roche Applied Sciences* (Mannheim, Germany). Concentration of DNA was calculated by Lambert-Berr law with the mean of the absorbtion measured by UV-Vis spectrometry at 260 nm. The molar extinction coefficients were given by the supplier. Water was purified by a MembraPure Milli-Q purification system from *Astacus* (Impedance 14.5 Ω).

Synthesis: Chemicals were purchased in either *puriss.*, *p.a.* or *purum* grade from *Fluka* (Steinheim, Germany), *Aldrich* (Steinheim, Germany), *Merck* (Darmstadt, Germany), *Acros* (Geel, Belgium), *Alfa-Aesar* (Karlsruhe, Germany) and *Lancaster* (Ward Hill, USA). Column chromatography was performed with *Merck* Silica Gel 60 (0.063 – 0.200 mm). TLC was performed with *Merck* Silica Gel 60 F254 plates. Ninhydrin and Seebach reagent was used as staining solution for UV inactive compounds. ^{1}H- and ^{13}C-NMR spectra were recorded with *Avance II-300* or *Avance II-500* spectrometer. The signals of the residual protonated solvent ([D_6]DMSO, $CDCl_3$, MeOD and [D_1]TFA) were used as reference signal. High-resolution mass spectra (HRMS) were measured with a LTQ FT- or LTQ FT Ultra-spectrometer from *Therma Finnigan*.

Solid-phase synthesis according to Boc/Cbz-strategy: Manual solid-phase synthesis was performed using 5 mL polyethylene syringe reactors from *MultiSynTech* equipped with a fritted disc and was performed at room temperature. The MBHA-resin (100 – 200 mesh, 1.2 mmol g^{-1}) was preloaded with Fmoc-Gly-OH or Boc-Lys(Fmoc)-OH (approx. 0.16 μmol mg^{-1}) according to standard protocols. The PNA conjugates were synthesized in 5 μmol scale by using standard Boc/Cbz-protected monomers. *Boc-cleavage*: The resin was treated with TFA/*m*-cresol (19:1, 2 × 5 min) and subsequently washed (10 × CH_2Cl_2, 10 × DMF). *Coupling*: 4 eq. building block (final concentration approx. 0.2 M), 4 eq. PyBOP and 8 eq. NMM in DMF was added to the resin, after 1 h shaking the resin was washed (5 × DMF, 5 × CH_2Cl_2, 5 × DMF). *Capping*: free amino groups were acetylated by treatment with 5 % Ac_2O, 6 % lutidine in DMF for 3 min followed by washing step (5 × DMF, 10 × CH_2Cl_2). After synthesis the crude PNA conjugate was cleaved from the MBHA resin (16:3:1, TFA:TFMSA:*m*-cresol, 0.5 mL, 2 h), extracted with TFA and the combined TFA phases precipitated in cold Et_2O (30 mL) and dissolved in H_2O.

Purification and characterization: The crude product was purified by semipreparative HPLC performed on an *Agilent 1100* series instrument (column: *Machery-Nagel* Nucleodur Gravity C18 250 × 21 5μ) using A (98.9 % H_2O, 1 % acetonitrile, 0.1 % TFA) and B (98.9 % acetonitrile, 1 % H_2O, 0.1 % TFA) as eluents in a linear gradient (3 – 30 % B in 30 min) with a flow rate of 15 mL min^{-1}, lyophilized and dissolved in H_2O. The product purity was analyzed by UPLC Acquity system from *Waters* (column: *Waters*Acquity UPLC BEH C18 1.7μ) in a linear gradient (3-30 % B in 2 min) with a flow rate of 0.5 mL·min^{-1} with eluent A (98.9 % H_2O, 1 % acetonitrile, 0.1 % TFA) and B (98.9 % acetonitrile, 1 % H_2O, 0.1 % TFA). Mass analysis was performed either with an ESI-MS instrument from *Agilent 1100* series (VL quadrupole in positive scan mode) with A (98.9 % H_2O, 1 % acetonitrile, 0.1 % formic acid) and B (98.9 % acetonitrile, 1 % H_2O, 0.1 % formic acid) with isocratic flow (50 % B) of 0.3 mL·min^{-1} in 2 min or with Maldi-Tof/MS using a Voyager-DE Pro Biospectrometry Workstation (*PerSeptive Biosystems*) and AXIMA Confidence (*Shimadzu Scientific Instruments*) with sinapinic acid as matrix. The collected fractions were lyophilized and stored at -20 °C.

Determination of yields: The yield was determined by measuring the optical density at 260 nm of a diluted aliquot of the PNA conjugate in degassed buffer (10 mM NaH_2PO_4, 150 mM NaCl, pH adjusted to 7.4) by using a quartz cuvette with 1 cm path length. The concentration of the PNA conjugate was determined by means of Lambert-Beer law. The molar extinction coefficient of each PNA conjugate at 260 nm was calculated using the following equation:[1, 2]

$$\varepsilon_{260\ nm} = \{(8.8 \times \#\ T) + (7.3 \times \#\ C) + (11.7 \times \#\ G) + (15.4 \times \#\ A)\} \times 0.9 \times 10^3\ M^{-1}cm^{-1}$$

In the equation, # represents the number nitrogenous base, whereupon T represents thymine, C represents cytosine, G represents guanine and A represents adenine.

Synthesis of aminoethyllysine cytidine building block (c*)

The synthesis of the PNA building block was partly adapted from procedures mentioned elsewhere.[3-5]

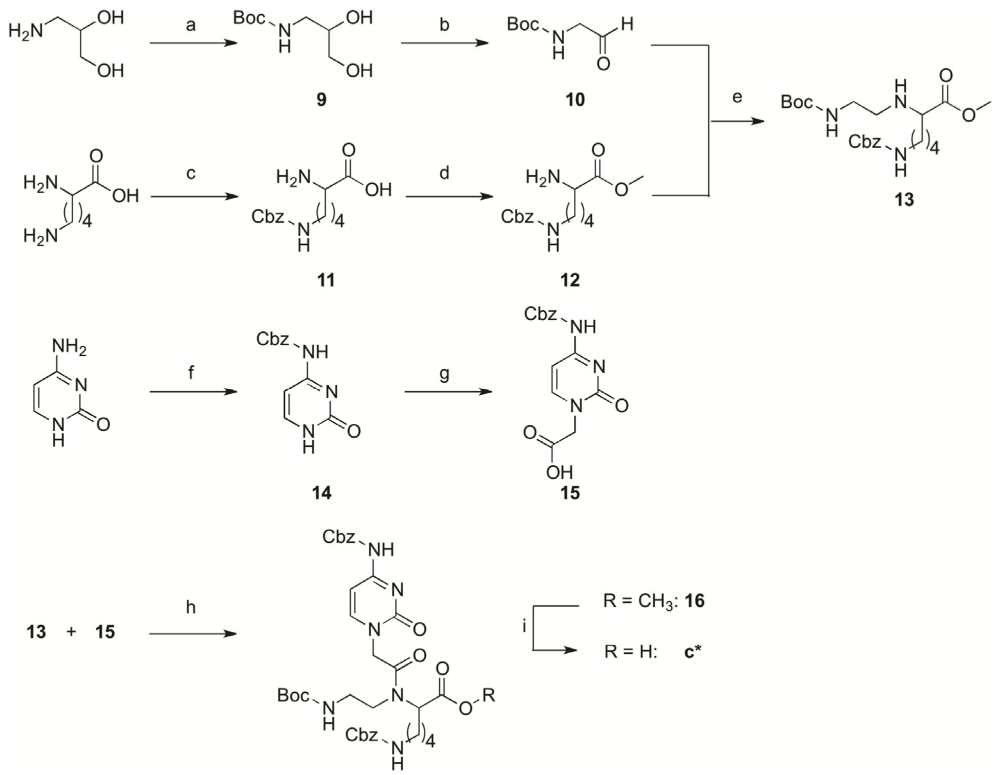

Figure S1: Synthesis of aminoethyllysine cytidine building block**c***; a) Boc_2O, EtOH, 70 %; b) $NaIO_4$, H_2O, 47 %; c) NaOH, $CuSO_4$, $NaHCO_3$, Cbz.-Cl, EDTA-Na, 70 %; d) MeOH, $SOCl_2$, quant.; e) MeOH, $NaCNBH_3$, 56 %; f) pyridine, Cbz-Cl, 46 %; g) K_2CO_3, $BrCH_2COOCH_3$, NaOH, 30 %; h) acetonitrile, DMF, *t*BuCOCl, 55 %; i) THF, LiOH, 72 %.

3-(*tert*-butyloxycarbonyl)aminopropane-1,2-diol (9)[6, 7]**:** To a solution of 3-aminopropane-1,2-diol (3.00 g, 33 mmol) in EtOH (50 mL) was added Boc_2O (8.60 g, 39.5 mmol). The reaction mixture was stirred at room temperature for 20 h. The solvent was evaporated under reduced pressure and the remaining solid

extracted with ethyl acetate and H_2O. The combined organic extracts were dried with anhydrous $MgSO_4$ and concentrated to yield 70 % (4.35 g, 23 mmol) of the product **9** as colourless oil.

R_f = 0.43 ($CHCl_3$/MeOH (9:1)); HRMS: *m/z* = 192.1230 ([M $^+$]: 192.1230); ^{1}H-NMR (300 MHz, $CDCl_3$, 25 °C): δ [ppm] = 5.38 (brs, 1H, N*H*), 3.94 – 3.73 (m, 1H, C*H*OH), 3.72 – 3.59 (m, 2H, CH_2OH), 3.57 – 3.18 (m, 2H, CH_2NH), 3.18 (s, 2H, 2 × O*H*), 1.42 (s, 9H, C(CH_3)$_3$); ^{13}C-NMR (75 MHz, $CDCl_3$, 25 °C): δ [ppm] = 157.17 (CO), 79.82 (C(CH_3)$_3$), 71.17 (CHOH), 63.52 (CH_2OH), 42.63 (CH_2NH), 28.21 (CH_3).

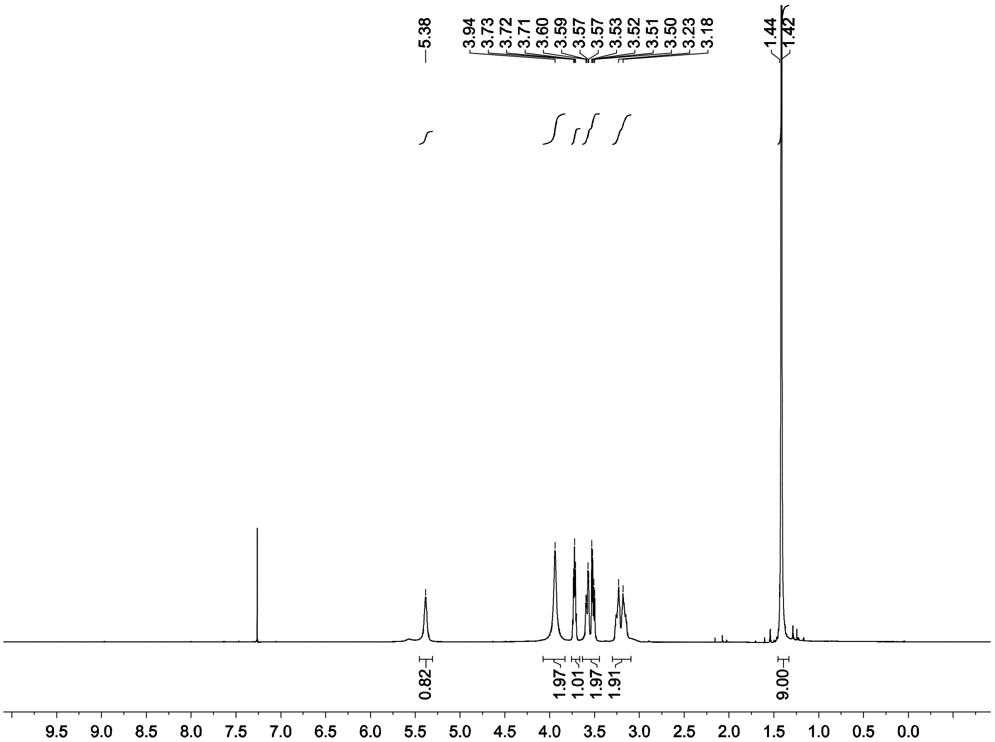

Figure S2. 1H-NMR spectrum of compound **9**.

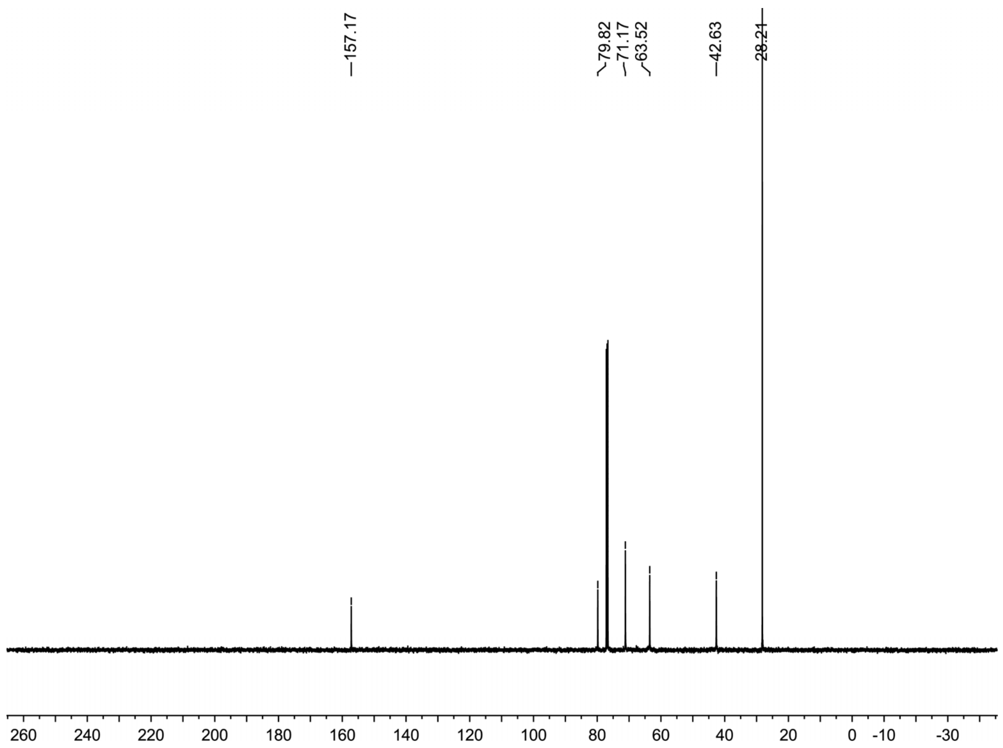

Figure S3. 13C-NMR spectrum of compound **9**.

3-(*tert*-butyloxycarbonyl)aminoacetaldehyde (10)[7]: To a solution of 3-(*tert*-butyloxycarbonyl)aminopropane-1,2-diol (**9**) (4.35 g, 23 mmol) in H_2O (40 mL) was added $NaIO_4$ (4.70 g, 23 mmol). The solution was stirred at room temperature for 1 h. The precipitate was filtered and the filtrate extracted with $CHCl_3$. The combined organic extracts were washed with brine and dried with anhydrous $MgSO_4$ and concentrated under reduced pressure to yield 47 % (1.67 g, 11 mmol) of product **10** as colourless oil.

R_f = 0.50 ($CHCl_3$/MeOH (9:1)); HRMS: *m/z* = 230.0788 ([M^+]: 230.0789); ^{1}H-NMR (300 MHz, [D_6]DMSO, 25 °C):δ [ppm] = 9.45 (s, 1H, C*H*O), 7.22 – 7.19 (t, ^3J (H,H) = 6 Hz, 1H, N*H*), 3.74 – 3.72 (d, ^3J (H,H) = 6 Hz, 2H, C*H₂*), 1.40 (s, 9H, C(C*H*$_3$)$_3$); ^{13}C-NMR (75 MHz, [D_6]DMSO, 25 °C): δ [ppm] = 201.12 (CHO), 146.42 (CO), 78.77 (C(CH_3)$_3$), 50.79 (CH_2NH), 28.57 (CH_3).

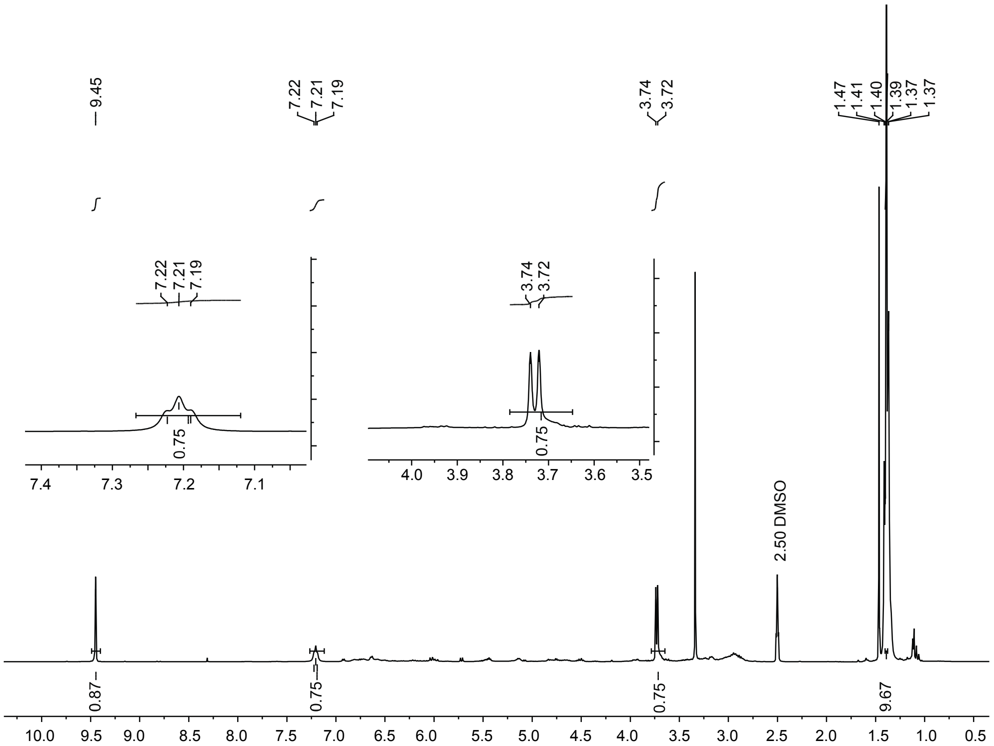

Figure S4. 1H-NMR spectrum of compound **10**.

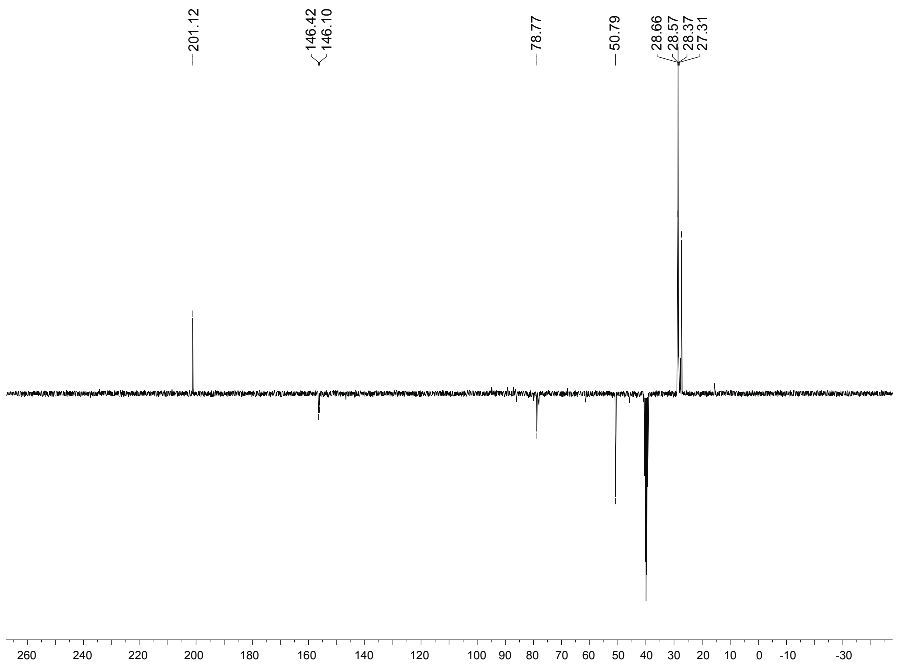

Figure S5. 13C-NMR spectrum of compound **10**.

***N*^ε^-(benzyloxycarbonyl)-L-lysine hydrochloride (11):** L-lysine hydrochloride (5.07 g, 27.8 mmol) and NaOH (2.31 g, 57.8 mmol) were dissolved in H_2O (20 mL). A solution of $CuSO_4$ × 5 H_2O (2.16 g, 13.7 mmol) in H_2O (15 mL) was added. After addition of $NaHCO_3$ (2.82 g, 33.5 mmol) the reaction mixture was cooled on ice bath followed by slow addition of benzylchloroformat (5.3 mL, 35.7 mmol). The reaction mixture was stirred for 20 h at room temperature. The blue precipitate was filtrated, washed with H_2O (50 mL), acetone (25 mL) and suspended in a prior made solution of EDTA (8.98 g, 30.8 mmol) and NaOH (2.53 g, 63.3 mmol) in H_2O (50 mL), the pH was adjusted to 7.0 with concentrated HCl. The reaction mixture was stirred for 20 h at room temperature subsequently filtrated and washed several times with H_2O. The white product **11**was dried under reduced pressure to yield 70 % (5.41 g, 19.3 mmol) of white product **11**.

MS: *m/z* = 281.14 ([M $^+$]: 281.30); ^{1}H-NMR (300 MHz, [D_1]TFA, 25 °C): δ [ppm] = 7.24 – 7.21 (m, 5H, C*H*-Cbz), 5.29 (s, 2H, OCH_2-Cbz), 4.28 – 4.19 (m, 1H, C*H*), 3.20 – 3.16 (m, 2H, NCH_2), 2.14 – 2.08 (m, 2H, CH_2), 1.82 – 1.81 (m, 2H, CH_2), 1.68 – 1.55 (m, 2H, CH_2); ^{13}C-NMR (75 MHz, [D_1]TFA, 25 °C): δ [ppm] = 174.56 (COOH), 134.33 (CO), 130.99 (C_q-Cbz), 130.31 (CH-Cbz), 130.07 (CH-Cbz), 129.35 (CH-Cbz), 126.79 (CH-Cbz), 72.74 (OCH_2-Cbz), 55.65 (CH), 42.35 (NCH_2), 30.92 (CH_2), 27.94 (CH_2), 23.26 (CH_2).

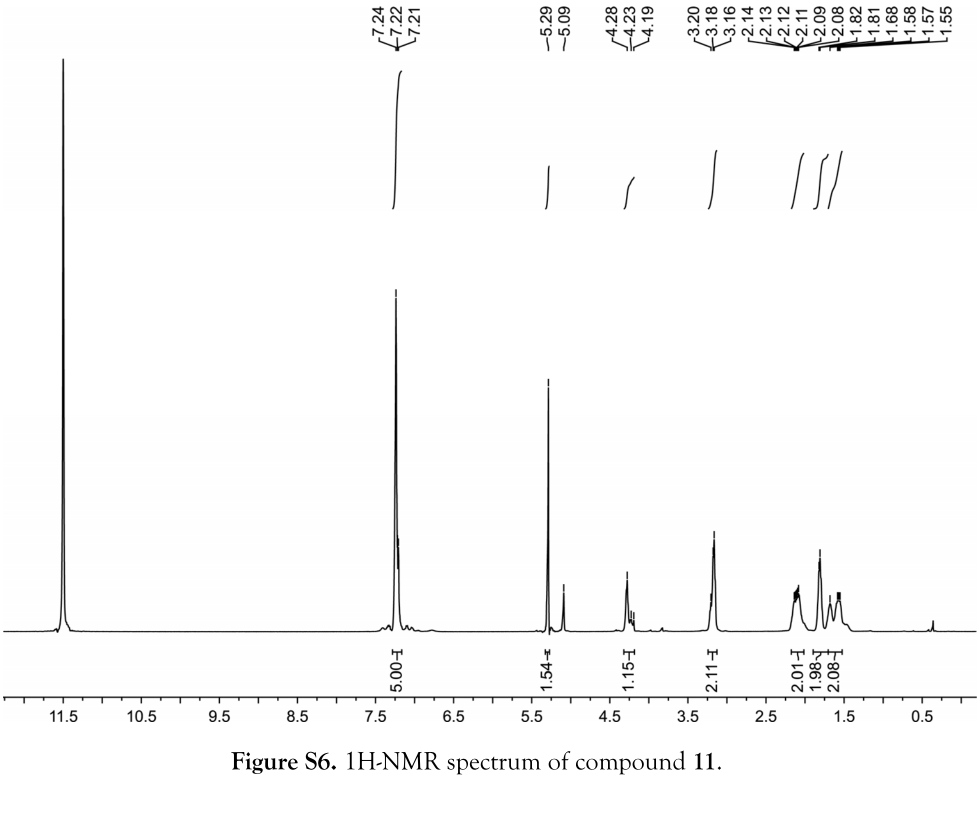

Figure S6. 1H-NMR spectrum of compound **11**.

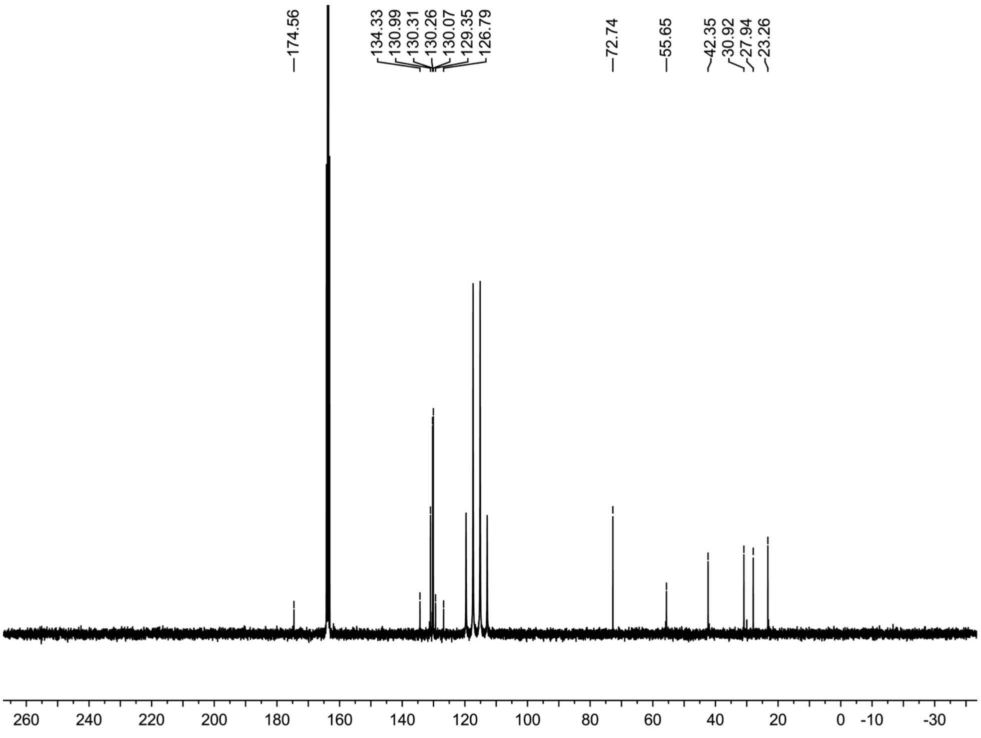

Figure S7. 13C-NMR spectrum of compound **11**.

N^{ε}-(benzyloxycarbonyl)-L-lysine methylester hydrochloride (12)[8]**:** N^{ε}-(benzyloxycarbonyl)-L-lysine (**11**) (10.00 g, 36 mmol) was suspended in MeOH (50 mL) and thionyl chloride (2.60 mL, 36 mmol) dropwise added under ice bath cooling. The reaction mixture was refluxed at 80 °C for 24 h. The solvent was removed under reduced pressure obtaining the yellow product **12** in 100 % (10.6 g, 36 mmol) yield.

HRMS: *m/z* = 295.1652 ([M$^+$]: 295.1652); ^{1}H-NMR (300 MHz, [D$_6$]DMSO, 25 °C): δ [ppm] = 8.50 (s, 2H, N*H$_2$*), 7.38 – 7.27 (m, 5H, C*H*-Cbz), 5.00 (s, 2H, OC*H$_2$*-Cbz), 4.00 (m, 1H, C*H*), 3.74 (s, 3H, OC*H$_3$*), 3.00 – 2.97 (d, ^3J (H,H) = 6 Hz, 2H, C*H$_2$*), 1.80 – 1.77 (d, ^3J (H,H) = 6 Hz, 2H, C*H$_2$*) 1.40 – 1.39 (m, 4H, 2 × C*H$_2$*); ^{13}C-NMR (75 MHz, [D$_6$]DMSO, 25 °C): δ [ppm] = 170.38 (COOMe), 156.28 (CO), 137.57 (C_q-Cbz), 128.69 (CH-Cbz), 128.07 (CH-Cbz), 65.47 (OCH_2-Cbz), 53.09 (OCH_3), 52.15 (CH), 38.35 (CH_2CH) 26.57 (CH_2), 21.81 (CH_2), 21.51 (CH_2).

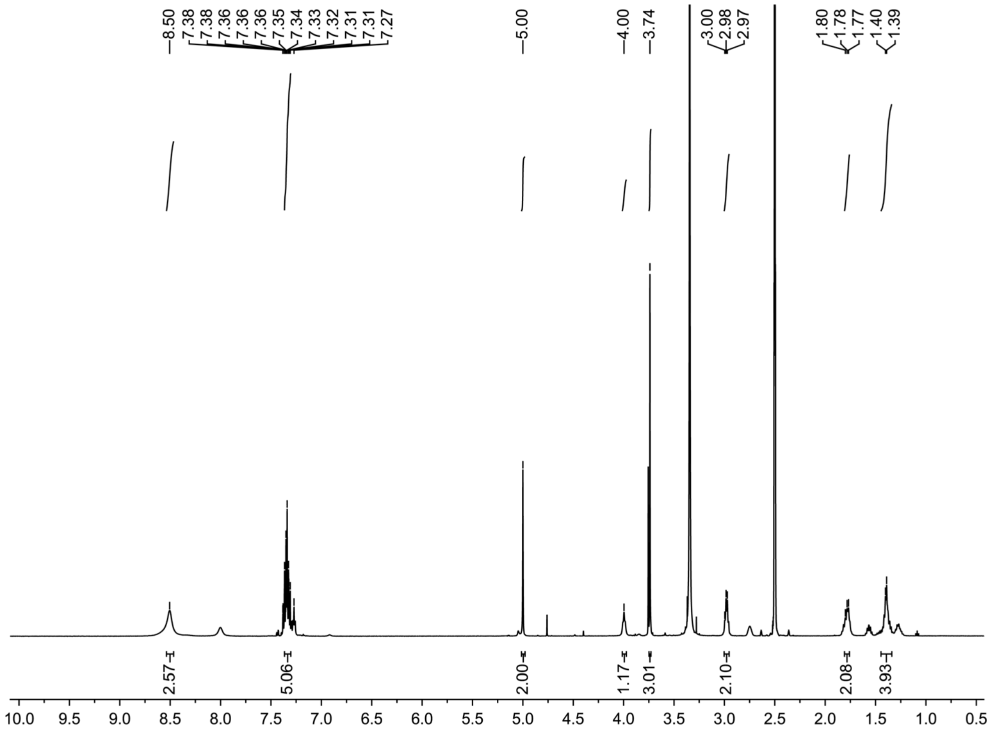

Figure S8. 1H-NMR spectrum of compound **12**.

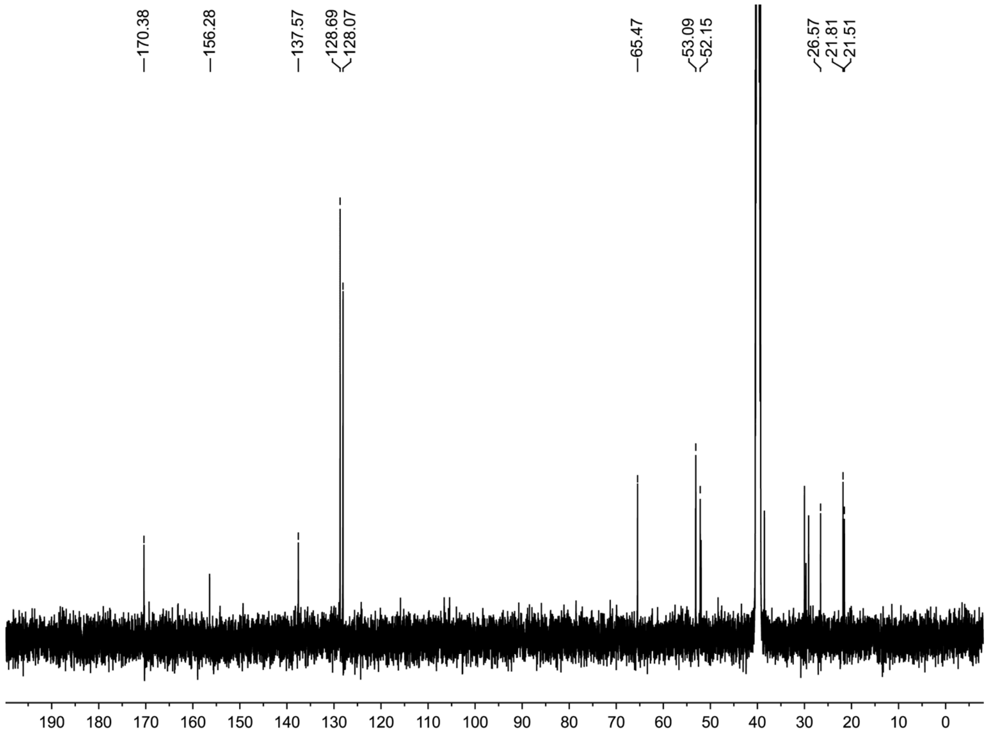

Figure S9. 13C-NMR spectrum of compound **12**.

N^{α}-(*tert*-butyloxycarbonyl)aminoethyl-N^{ε}-(benzyloxycarbonyl)-L-lysine methylester (13): N^{ε}-(benzyloxycarbonyl)-L-lysine methylester (**12**) (1.85 g, 6.30 mmol) was dissolved in MeOH (10 mL) and added to a solution of 3-(*tert*-butyloxycarbonyl)aminoacetaldehyde (**5**) (1.07 g, 6.30 mmol) in MeOH (30 mL). The reaction mixture was cooled on an ice bath and stirred for 30 min before acetic acid (0.37 mL) and $NaCNBH_3$ (0.22 g, 3.5 mmol) were added. The reaction mixture was stirred for 20 h at room temperature. After evaporation of the solvent the crude product was purified by flash column chromatography (cyclohexane/ethyl acetate, 1:5) to yield 56 % (1.54 g, 3.5 mmol) of the product **13**as yellow oil.

R_f = 0.30 ($CHCl_3$/MeOH (9:1)); HRMS: *m/z* = 438.2597 ([M^+]: 438.2599); ^{1}H-NMR (300 MHz, [D_6]DMSO, 25 °C): δ [ppm] = 7.36 – 7.34 (m, 5H, C*H*-Cbz), 7.34 – 7.31 (t, ^3J (H,H) = 5 Hz, 1H, N*H*), 6.71 (t, ^3J (H,H) = 5 Hz, 1H, N*H*), 5.00 (s, 2H, OC*H$_2$*-Cbz), 3.62 (s, 3H, OC*H$_3$*), 3.33 – 3.14 (t, ^3J (H,H) = 5 Hz, 1H, C*H*), 2.98 – 2.94 (m, 4H, 2 × C*H$_2$*), 2.52 – 2.50 (m, 2H, NC*H$_2$*), 1.49 – 1.39 (m, 2H, C*H$_2$*) 1.37 (s, 9H, C(C*H$_3$*)$_3$), 1.27 – 1.25 (m, 2H, C*H$_2$*); ^{13}C-NMR (75 MHz, [D_6]DMSO, 25 °C): δ [ppm] = 175.17 (COOMe), 156.03 (CONH), 155.58 (COtBu), 137.27 (C_q-Cbz), 128.68 (CH-Cbz), 128.29 (CH-Cbz), 77.44 (Cq-*t*Bu), 65.05 (OCH_2-Cbz), 60.56 (CH_2NH), 59.72 (CH), 51.23 (OCH_3), 47.10 (CH_2N), 32.35 (CH_2), 29.20 (CH_2), 28.20 (CH_3), 22.55 (CH_2).

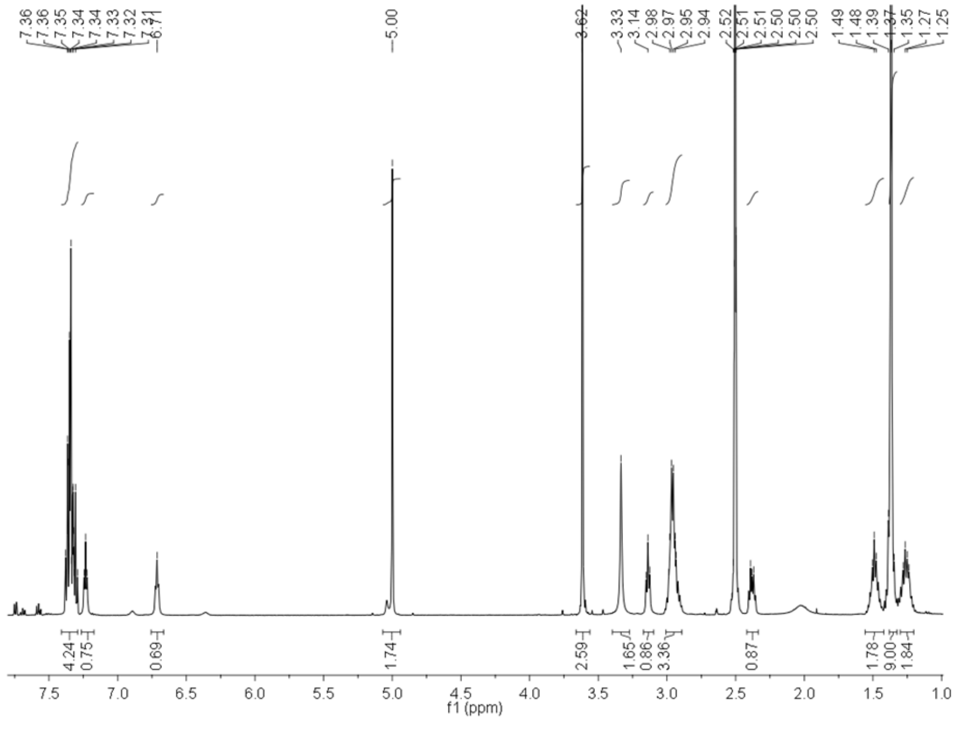

Figure S10. 1H-NMR spectrum of compound **13**.

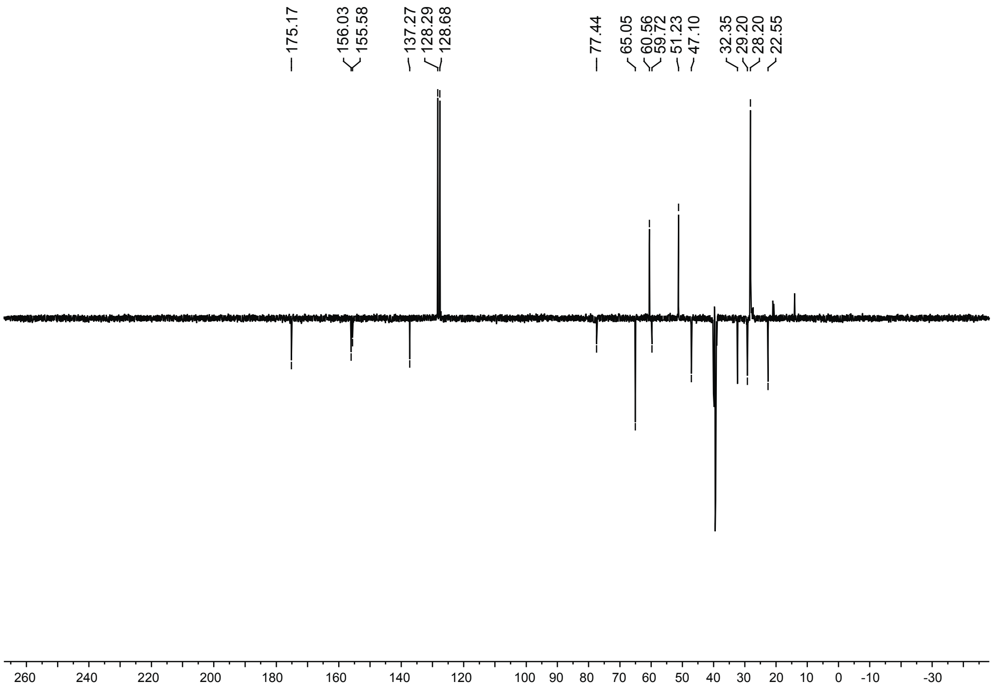

Figure S11. 13C-NMR spectrum of compound **13**.

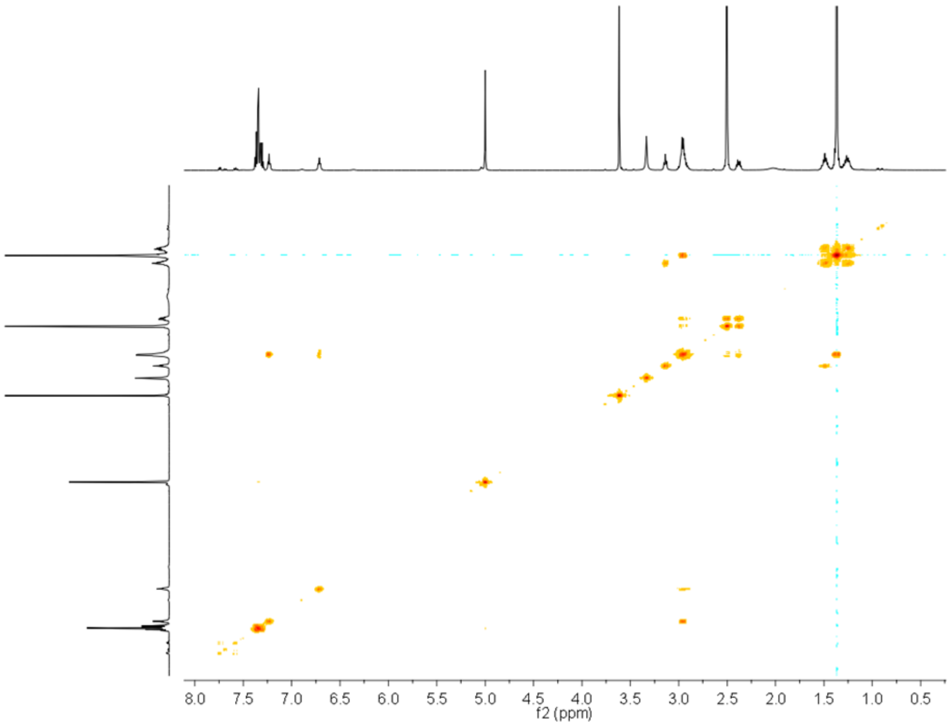

Figure S12. 2D-NMR (HH-COSY) spectrum of compound **13**.

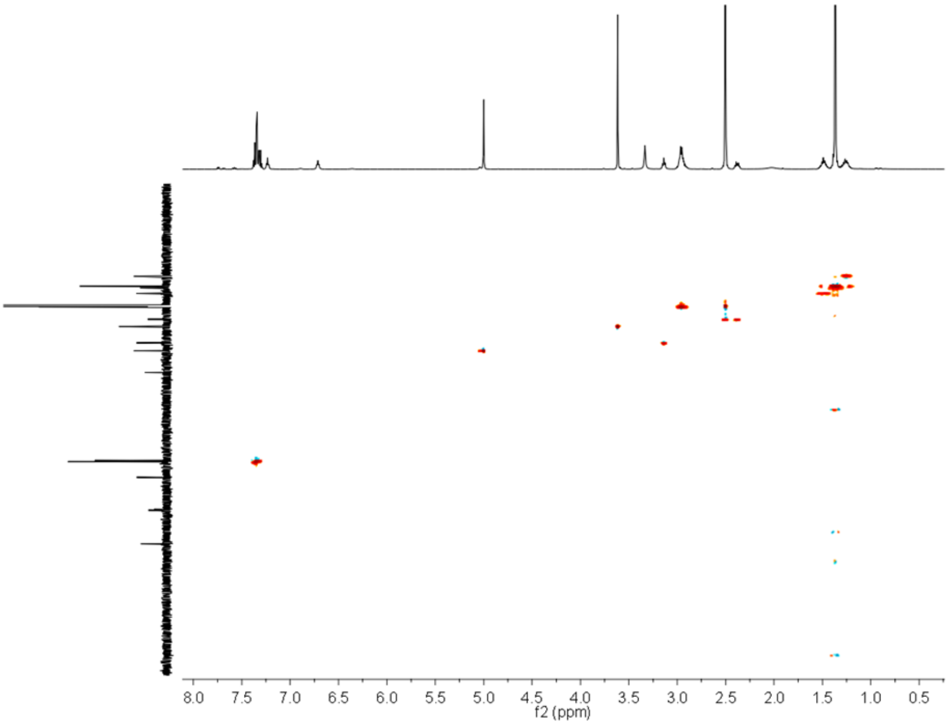

Figure S13. 2D-NMR (HSQC) spectrum of compound **13**.

***N*⁴-(benzyloxycarbonyl)cytosine (14):** Benzylchloroformate (14 mL, 90.0 mmol) was added dropwise to a cooled (ice bath) suspension of cytosine (5.00 g, 45.0 mmol) in dry pyridine (200 mL . The reaction mixture was stirred for 24 h at room temperature. The solvent was removed under reduced pressure. The crude product was suspended in H_2O (50 mL) and the pH adjusted to pH 1 with concentrated HCl. The precipitated product was collected by filtration and dried under reduced pressure to yield 46 % of **14** (5.10 g, 20.8 mmol) as white product.

HRMS: *m/z* = 246.0871 ([M^+]: 246.0873); ^{1}H-NMR (300 MHz, [D_6]DMSO, 25 °C): δ [ppm] = 7.81 – 7.79 (d, 1H, ^3J (H,H) = 5 Hz, C*H*), 7.41 – 7.35 (m, 5H, C*H*-Cbz), 6.93– 6.92 (d, 1H, ^3J (H,H) = 5 Hz, C*H*), 5.18 (s, 2H, OCH_2-Cbz), 3.33 (s, 1H, N*H*); ^{13}C-NMR (75 MHz, [D_6]DMSO, 25 °C): δ [ppm] = 163.61 (CO), 146.69 (CH), 136.00 (CO), 128.44 (CH-Cbz), 128.09 (CH-Cbz), 127.87 (CH-Cbz), 93.47 (CH), 66.36 (OCH_2).

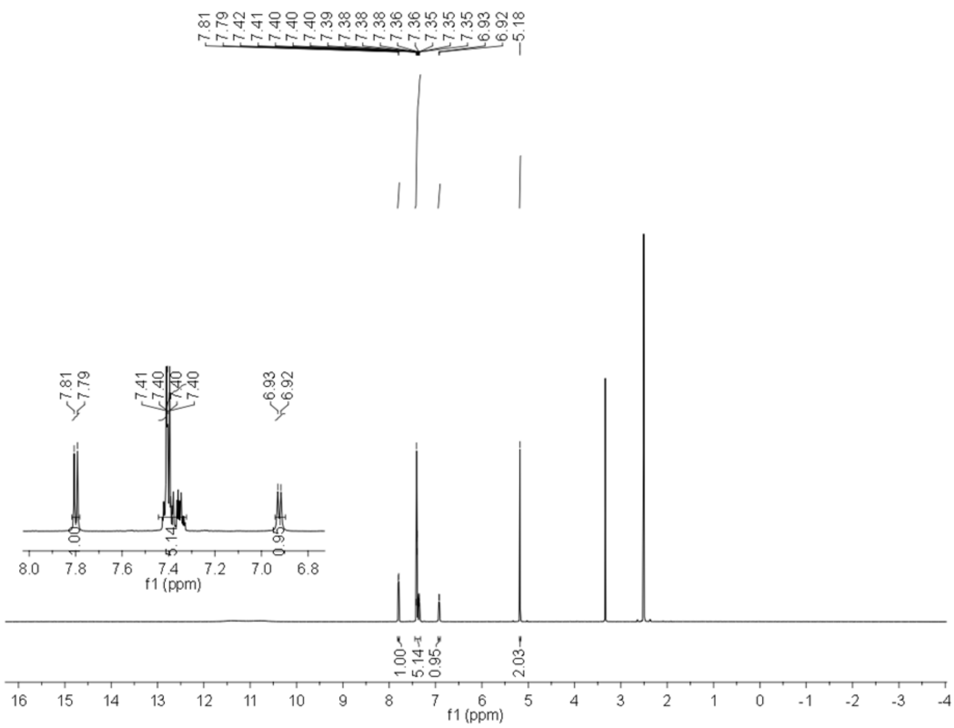

Figure S14. 1H-NMR spectrum of compound **14**.

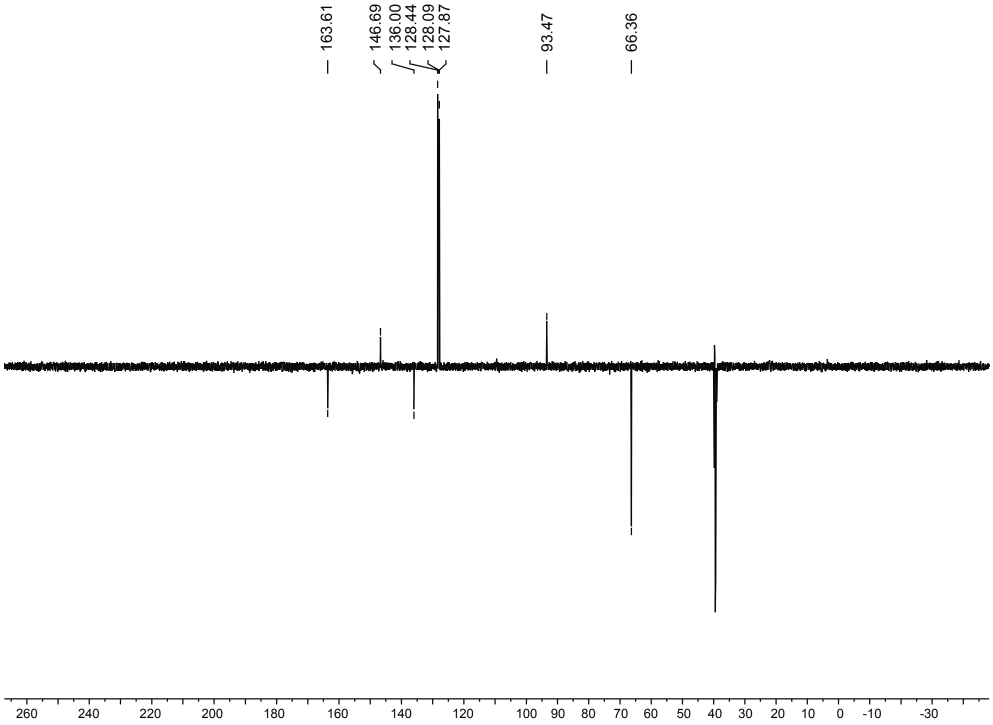

Figure S15. 13C-NMR spectrum of compound **14**.

N^4-(benzyloxycarbonyl)cytosine-1-ylacetic acid (15): N^4-(benzyloxycarbonyl)cytosine (**14**) (8.37 g, 34.1 mmol) and K_2CO_3 (4.74 g, 34.1 mmol) were suspended in DMF (80 mL). Subsequently, methylbromoacetate (3.30 mL, 34.1 mmol) was added dropwise and the reaction mixture stirred for 48 h at room temperature. The precipitate was filtered and the filtrate concentratied to dryness under reduced pressure. The obtained solid was dissolved in H_2O (40 mL) and acidified with HCl (4 M) until the product precipitated. The solid was collected by filtration and subsequently dissolved in aqueous NaOH (50 mL, 1 M) and H_2O (25 mL) and stirred for 30 min at room temperature. The reaction mixture was cooled on an ice bath and filtered. The filtrate was acidified with HCl (4 M) until precipitation was complete. The white solid was filtered and dried under reduced pressure to yield 30 % (3.09 g, 10.1 mmol) of the product **15**.

HRMS: m/z = 304.0927 ([M^+]: 304.0928); ^{1}H-NMR (500 MHz, [D_6]DMSO, 25 °C): δ [ppm] = 10.83 (brs, 1H, C^4NH), 7.81– 7.79 (d, ^3J (H,H) = 5 Hz, 1H, C*H*), 7.43 – 7.35 (m, 5H, C*H*-Cbz), 7.03 – 7.02 (d, ^3J (H,H) = 5 Hz, 1H, C*H*), 5.19 (s, 2H, OCH_2-Cbz), 4.35 (s, 3H, CH_2COOH); ^{13}C-NMR (75 MHz, [D_6]DMSO, 25 °C): δ [ppm] = 169.36 (CO), 163.30 (C^2O), 146.68 (C^6H), 136.95 (CO), 128.49 (CH), 128.16 (CH), 127.94 (CH), 94.00 (C^5H), 66.51 (OCH_2), 50.52 (CH_2COOH).

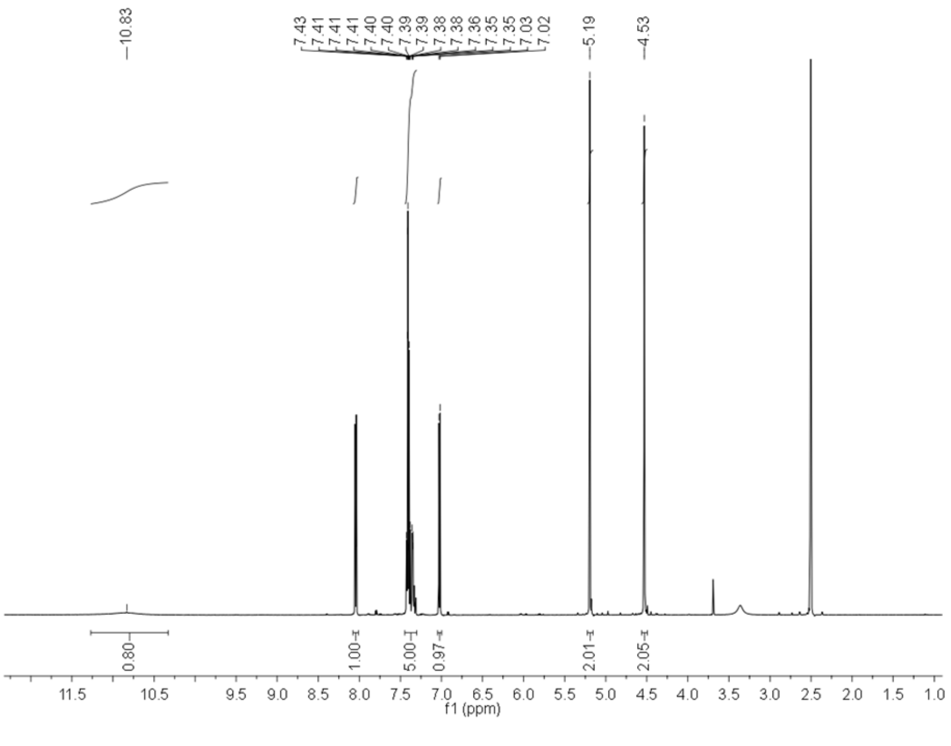

Figure S16. 1H-NMR spectrum of compound **15**.

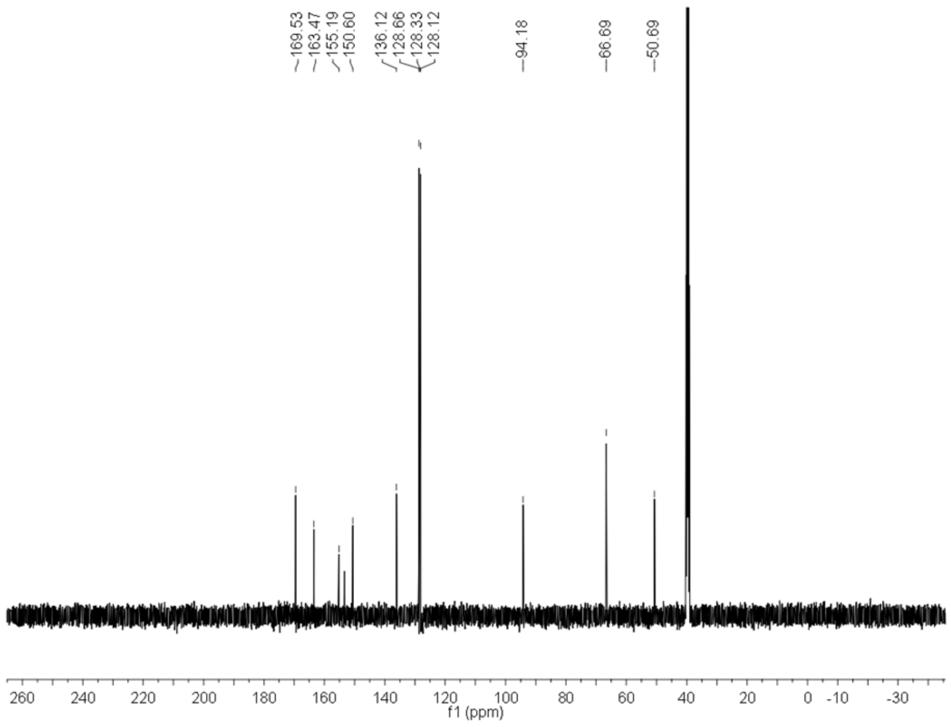

Figure S17. 13C-NMR spectrum of compound **15**.

N^{α}-((N^{4}-benzyloxycarbonyl)cytosine-1-yl)acetyl)-*N*-(2-*tert*-butyloxycarbonyl)aminoethyl)-N^{ε}-(benzyloxycarbonyl)-L-lysine methylester (16): N^4-((benzyloxycarbonyl)cytosine-1-yl)acetic acid (**15**) (1.27 g, 4.18 mmol) was suspended at -15 °C (MeOH/ice) in acetonitrile/DMF (1:1, 80 mL) and stirred for 5 min. Subsequent, pivaloyl chloride (673 µL, 5.1 mmol) was added and the reaction mixture stirred at -15 °C for further 15 min. Afterwards, N^{α}-(*tert*-butyloxycarbonyl)aminoethyl-N^{ε}-(benzyloxycarbonyl)-L-lysine methylester (**13**) (1.22 g, 2.80 mmol) dissolved in acetonitrile/DMF (1:1, 20 mL) was added dropwise and the reaction mixture stirred for 20 h at room temperature. After the reaction was complete the crude mixture was diluted with ethyl acetate (300 mL) and extracted with HCl (0.1 M, 3 × 100 mL). The combinded organic phases were dried with anhydrous $MgSO_4$ and purified by flash column chromatography (ethyle acetat) to yield 55 % (1.11 g, 1.50 mmol) of the product **16**.

R_f = 0.30 (ethyle acetate); HRMS: *m/z* = 723.3343 ([M^+]: 723.3348); ^{1}H-NMR (300 MHz, [D_6]DMSO, 25 °C): δ [ppm] = 7.63 – 7.53 (m, ^3J (H,H) = 5 Hz, 1H, C*H*), 7.38 – 7.23 (m, 10H, 2 × C*H*-Cbz) 7.22 (d, ^3J (H,H) = 6 Hz, 1H, C*H*), 5.49 (brs, 1H, N*H*), 5.40 (brs, 1H, N*H*), 5.21 (s, 2H, OCH_2-Cbz), 5.08 (s, 2H, OCH_2-Cbz), 4.77 – 4.52 (m, 2H, CH_2CH), 4.13 – 4.10 (t, ^3J (H,H) = 6 Hz, 1H, C*H*), 3.71 (s, 3H, OCH_3), 3.37 – 3.31 (m, 2H, NCH_2), 3.30 – 3.29 (m, 2H, CH_2-NBoc), 3.18 – 3.16 (m, 2H, CH_2-NCbz), 1.55 – 1.52 (m, 4H, 2 × CH_2), 1.43 (s, 9H, C(CH_3)$_3$), 1.33 – 1.31 (m, 2H, CH_2); ^{13}C-NMR (75 MHz, [D_6]DMSO, 25 °C): δ [ppm] = 175.17 (COOMe), 167.51 (CO), 163.53 (C_q), 156.03 (CONH), 155.58 (CO-*t*Bu), 137.27

(C_q-Cbz), 128.29 (CH), 127.68 (CH-Cbz), 77.44 (C_q-*t*Bu), 60.05 (OCH_2-Cbz), 59.72 (CH), 51.23 (OCH_3), 47.10 (CH_2N-*t*Bu), 32.35 (CH_2N-Cbz), 29.20 (CH_2), 28.20 ($(CH_3)_3$), 22.55 (CH_2).

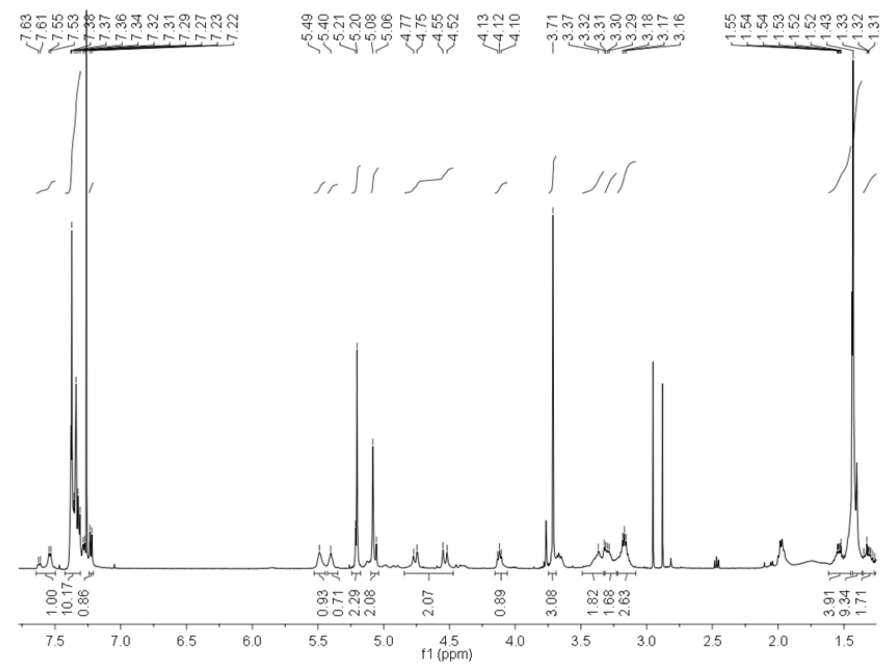

Figure S18. 1H – NMR spectrum of compound **16**.

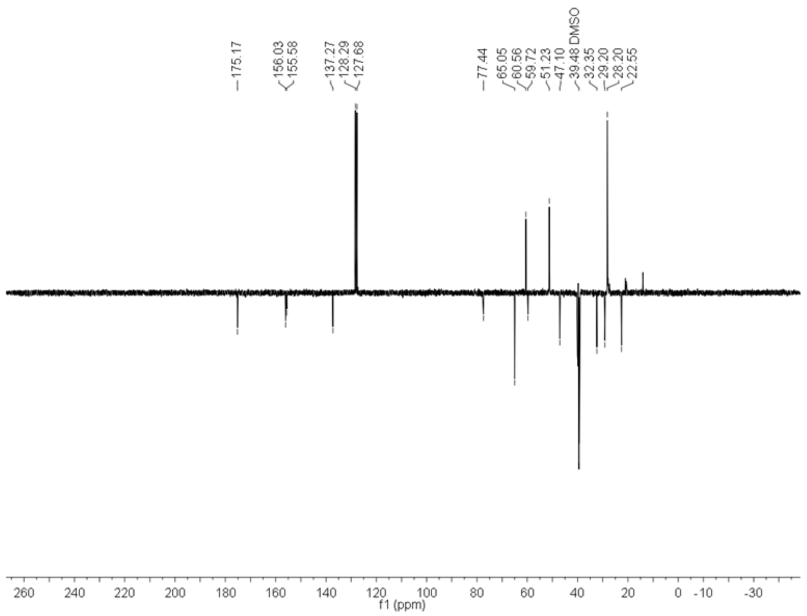

Figure S18. 13C - NMR spectrum of compound **16**.

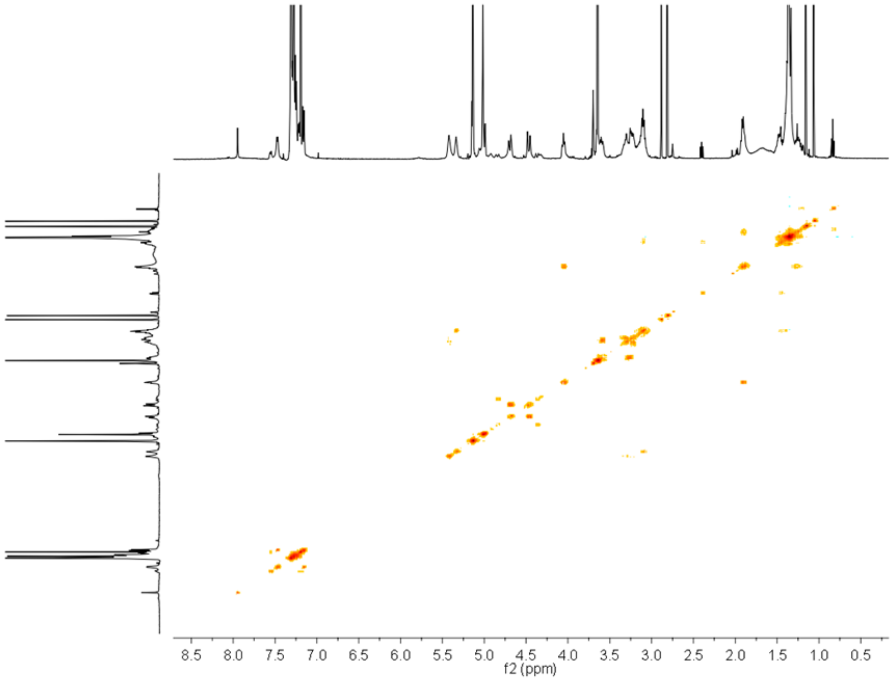

Figure S19. 2D – NMR (HH-COSY) spectrum of compound **16**.

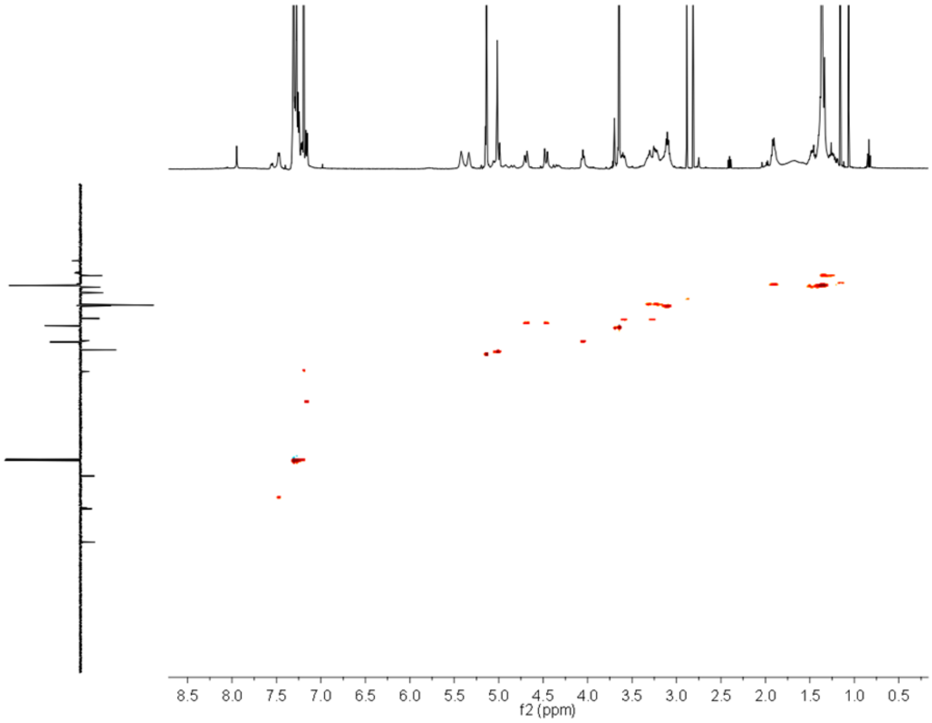

Figure S20. 2D-NMR (HSQC) spectrum of compound **16**.

N^{α}-((N^{4}-benzyloxycarbonyl)cytosine-1-yl)acetyl)-N-(2-*tert*-butyloxycarbonyl)aminoethyl)-N^{ε}-(benzyloxycarbonyl)-L-lysine (c*): To a cooled (ice bath) solution of **16** (53 mg, 0.71 mmol) in THF (5 mL) was added an aqueous solution of LiOH (2 mL, 1 M). The mixture was stirred for 3 h at room temperature. The volatiles were removed at reduced pressure, H_2O (10 mL) was added and the pH adjusted with HCl (1 M) to pH 3.0. The product was extracted with ethyl acetate, dried with anhydrous $MgSO_4$ and purified by flash column chromatography (ethyl acetate/MeOH/ formic acid, 95:5:1). The purified product was washed with HCl (0.1 M), dried with anhydrous $MgSO_4$ to yield 72 % (356 mg, 0.51 mmol) of aminoethyllysine cytidine building block (**c***).

R_f = 0.76 (ethyle acetate/MeOH/formic acid, 95:5:1); HRMS: *m/z* = 709.3194 ([M^+]: 709.3192); ^{1}H-NMR (500 MHz, [D_6]DMSO, 25 °C): δ [ppm] = 10.82 (brs, 1H, COO*H*), 7.96 – 7.95 (d, ^3J (H,H) = 5 Hz, 1H, C*H*), 7.41 – 7.38 (m, 5H, C*H*-Cbz), 7.36 – 7.34 (m, 5H, C*H*-Cbz), 7.26 (brs, 1H, N*H*), 7.03 – 7.01 (d, ^3J (H,H) = 5 Hz, 1H, C*H*), 7.01 (d, ^3J (H,H) = 5 Hz, 1H, C*H*), 5.19 (s, 2H, OCH_2-Cbz), 5.00 (s, 2H, OCH_2-Cbz), 4.97 (s, 2H, CH_2), 4.81 – 4.34 (m, 1H, C*H*), 3.35 – 2.73 (m, 6H, NCH_2), 1.35 (s, 9H, C(CH_3)$_3$), 1.91 – 1.17 (m, 6H, CH_2); ^{13}C-NMR (75 MHz, [D_6]DMSO, 25 °C): δ [ppm] = 172.36 (COOH), 167.43 (CO), 163.36 (CONH), 156.26 (CONH), 155.89 (CO-*t*Bu), 151.17 (CH), 137.47 (C_q-Cbz), 136.15 (C_q-Cbz), 128.65 (CH-Cbz), 128.51 (CH-Cbz), 128.31 (CH-Cbz), 128.09 (CH-Cbz), 127.89 (CH-Cbz), 93.97 (CH), 70.22 (C_q-*t*Bu), 66.65 (OCH_2-Cbz), 65.28 (OCH_2-Cbz), 60.43 (CH), 59.03 (CH_2CH), 57.21 (CH_2N-*t*Bu), 33.70 (CH_2N-Cbz), 29.34 (CH_2N), 28.44 (CH_2), 28.35 ((CH_3)$_3$), 23.31 (CH_2).

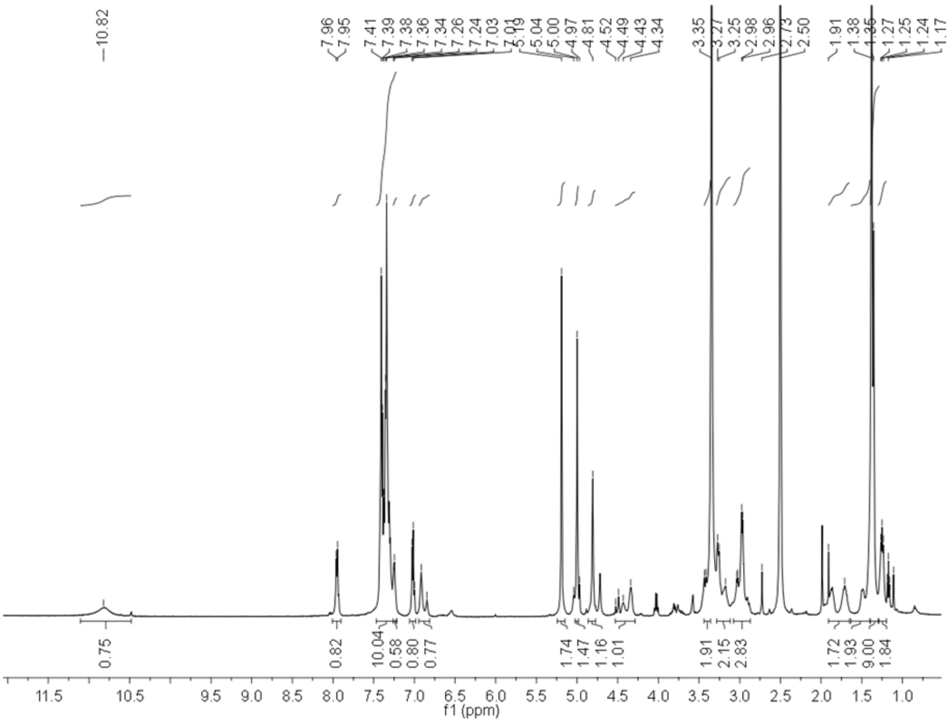

Figure S21. 1H-NMR spectrum of compound **c***.

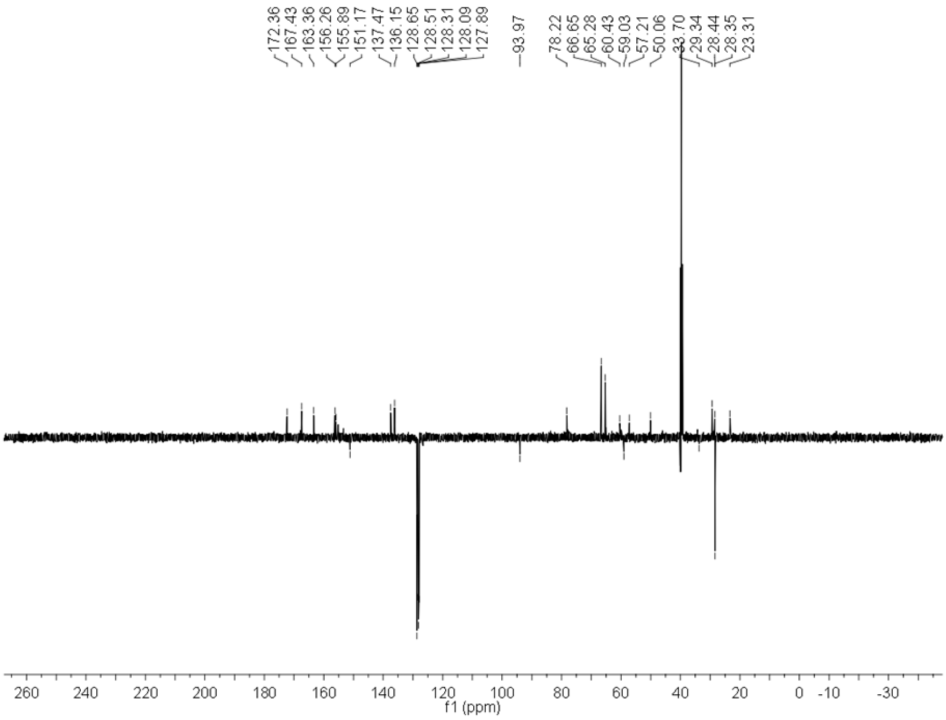

Figure S22. 13C-NMR spectrum of compound **c***.

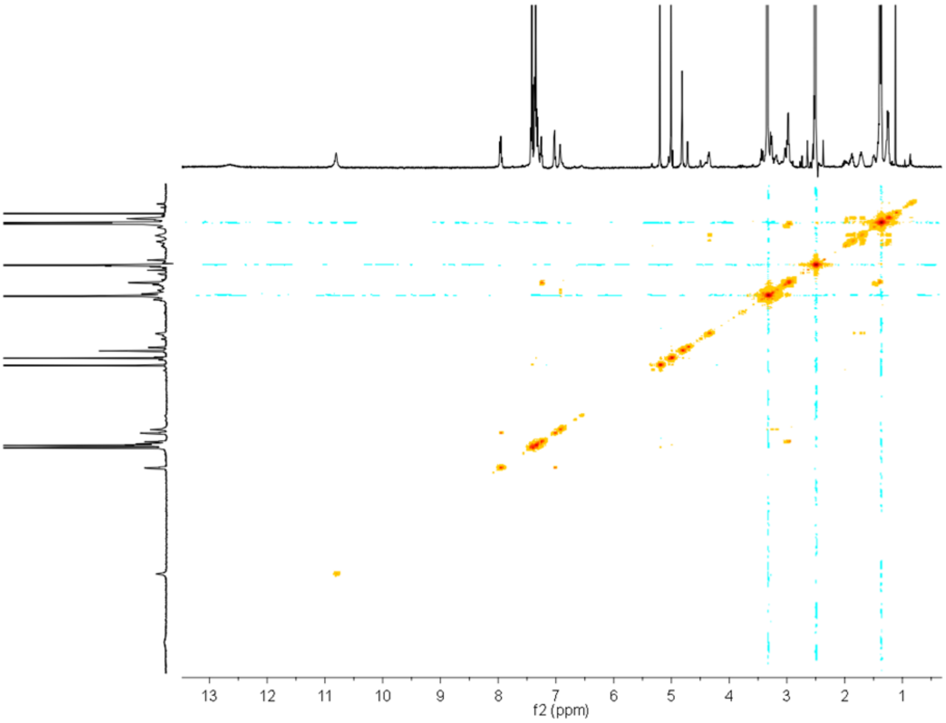

Figure S23. 2D-NMR (HH-COSY) spectrum of compound **c***.

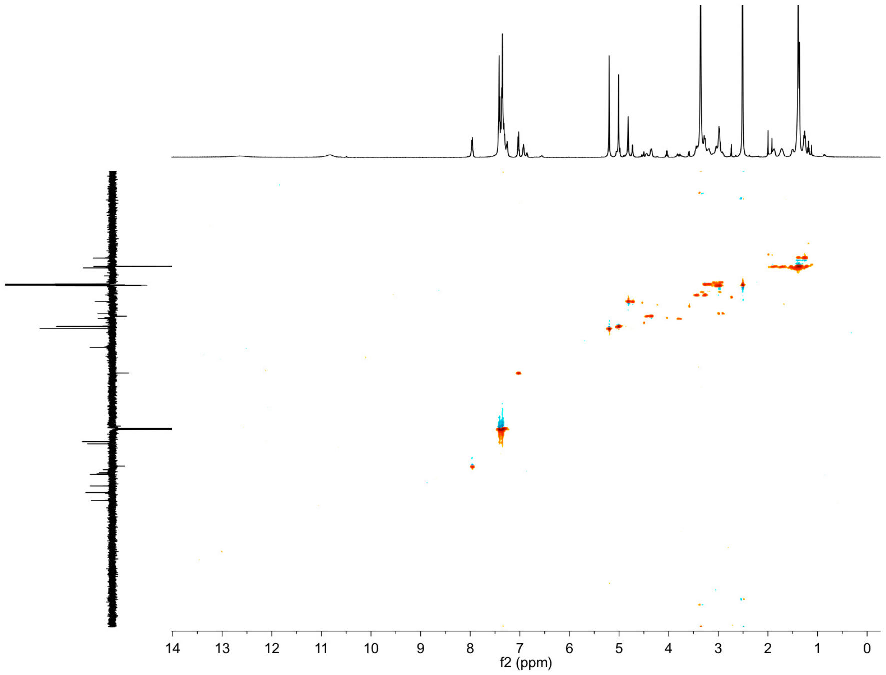

Figure S24. 2D-NMR (HSQC) spectrum of compound **c***.

Synthesis of PNA conjugates

The PNA oligomers, both the non-modified and the aminoethyllysine modified PNA oligomers, with *N*-terminal cysteine and C-terminal thioester moieties were synthesized on the solid phase by using Boc-chemistry on Fmoc-Gly or Fmoc-Lys derivatized MBHA resin. The C-terminal thioester moiety was assembled by coupling of S(Trt)-mercaptopropionic acid on Fmoc-Gly derivatized MBHA resin, the trityl group was removed upon treatment with trifluoroacetic acid (TFA) and triisopropylsilane (TIS) (19:1, v:v), followed by coupling of Boc-beta-alanine. The linear PNA synthesis was carried out by using PyBOP (4 equiv.) and NMM (8 equiv.) as coupling reagent in DMF. The deprotection of Cbz-protecting group on the side-chain amine and exocyclic amine was achieved simultaneously during the final step of cleavage of the PNA oligomer from the resin using TFA, trifluoromethanesulfonic acid (TFMSA) and *m*-cresol as scavenger in the ratio 16:3:1. The crude product was precipitated in cold diethyl ether and cooling with dry ice. The precipitate was collected by decanting. After drying with argon the precipitate was dissolved in water (0.1 % TFA) and purified by reversed-phase (RP) HPLC. The final purity was checked by analytical RP-UPLC (C18). The identity and purity of products was assessed by ESI-MS or MALDI-TOF. For ESI-MS: Values are given in mass-to-charge ratio (m/z). Molecular weights were calculated from the peak set of the corresponding multiple charged ions using the deconvolution tool of *Chemstation.* Ligation products, for melting curve measurements, were synthesised by reaction of corresponding reactive PNA probes for 24 h in 180 μM concentration in 200 mM NaH_2PO_4, 50 mM MesNa, pH 7.4 and purified by RP-HPLC. To transform the thioester moiety of the mercaptopropionic thioester into the more reactive mercaptoethanesulfonic acid thioester the mercaptopropionic thioester was incubated for 3 h in 150 mM NaH_2PO_4, 30 mM MesNa, pH 7.4 and subsequently purified by RP-HPLC.

1a: Ac-cagcag-βAla-S(CH_2)$_2$CO-Gly; OD_{260} =56.3; 2645 nmol; 26 % yield;ε_{260} = 57800 L·mol^{-1}·cm^{-1}; t_R = 0.69 min; Formula: $C_{74}H_{95}N_{41}O_{20}S$, ESI-MS: $[M+5H]^{5+}$: 383.0 (calcd. = 383.0); $[M+4H]^{4+}$: 478.1 (calcd. = 478.4); $[M+3H]^{3+}$: 637.3 (calcd. = 637.6); $[M+2H]^{2+}$: 956.0 (calcd. = 955.9); MW_{dec} = 1910.77 (calcd. = 1910.87).

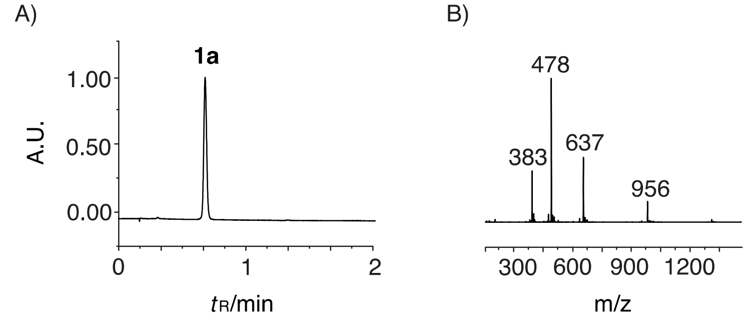

Figure S25. A) UPLC and B) ESI-MS analysis of purified compound **1a**.

1b: Ac-cagcag-βAla-S(CH_2)SO_3H; OD_{260} = 7.94; 186 nmol; 56 % yield ; ε_{260} = 57800 L·mol^{-1}·cm^{-1}; t_R = 1.51 min; Formula: $C_{71}H_{90}N_{39}O_{21}S_2$, ESI-MS: $[M+4H]^{4+}$: 472.9 (calcd. = 473.2); $[M+3H]^{3+}$: 630.2 (calcd. = 630.6); $[M+2H]^{2+}$: 956.0 (calcd. = 945.3); MW_{dec} = 1889.75 (calcd. = 1889.85).

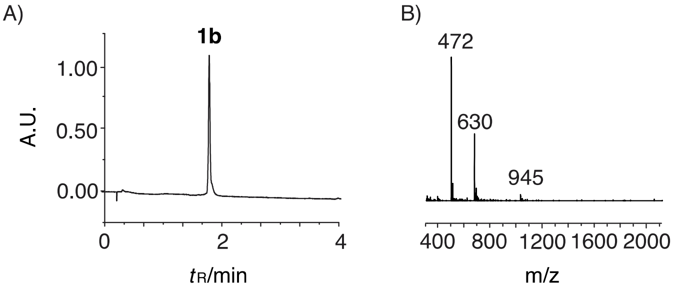

Figure S26. A) HPLC and B) ESI-MS analysis of purified compound **1b**.

2: Cys-cagcag-Gly; OD_{260} = 2.14; 146 nmol; 12 % yield; ε_{260} = 57800 L·mol^{-1}·cm^{-1}; t_R = 0.67 min; Formula: $C_{69}H_{89}N_{41}O_{18}S$, ESI-MS: $[M+5H]^{5+}$: 354.1 (calcd. = 363.3); $[M+4H]^{4+}$: 452.8 (calcd. = 453.9); $[M+3H]^{3+}$: 605.2 (calcd. = 604.9); $[M+2H]^{2+}$: 956.0 (calcd. = 906.9); MW_{dec} = 1812.34 (calcd. = 1812.77).

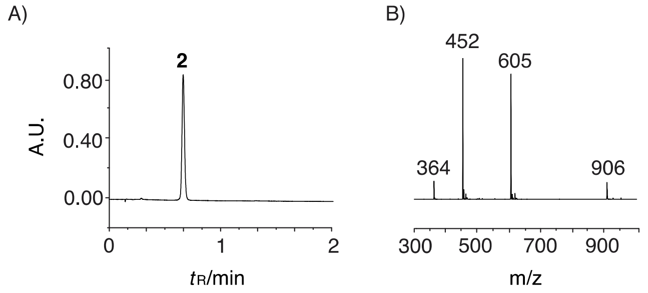

Figure S27. A) HPLC and B) ESI-MS analysis of purified compound **2**.

3: Ac-cagcag-βAlaCys-cagcag-Gly; OD_{260} = 0.15; 120 pmol; 42 % yield (peak area); ε_{260} = 127600 L·mol^{-1} ·cm^{-1}; t_R = 1.62 min; Formula: $C_{138}H_{174}N_{80}O_{36}S$, ESI-MS:$[M+7H]^{7+}$: 508.9 (calcd. = 509.5); $[M+6H]^{6+}$:

594.2 (calcd. = 594.0); $[M+5H]^{5+}$:713.8 (calcd. = 712.9); $[M+4H]^{4+}$: 891.3(calcd. = 890.9); $[M+3H]^{3+}$: 1187.6 (calcd. = 1187.5); MW_{dec} = 3561.61 (calcd. = 3561.43).

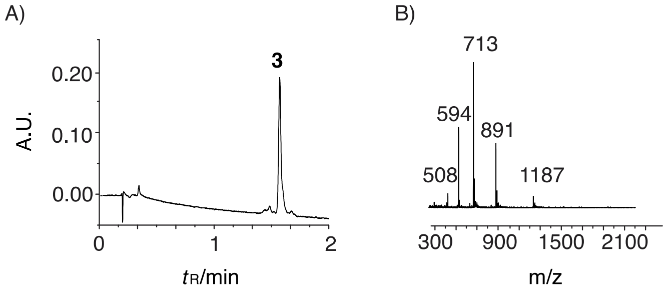

Figure S28. A) HPLC and B) ESI-MS analysis of purified compound **3**.

4a: Ac-c*agc*ag-βAla- $S(CH_2)_2CO$-Gly; OD_{260} = 635.6; 664 nmol; 26 % yield; ε_{260} = 57800 L·mol^{-1}·cm^{-1}; t_R = 1.51 min; Formula: $C_{79}H_{108}N_{41}O_{21}S_2$, ESI-MS: $[M+5H]^{5+}$: 411.6 (calcd. = 411.6); $[M+4H]^{4+}$: 514.3 (calcd. = 514.3); $[M+3H]^{3+}$: 685.2 (calcd. = 685.4); MW_{dec} = 2053.04 (calcd. = 2053.11).

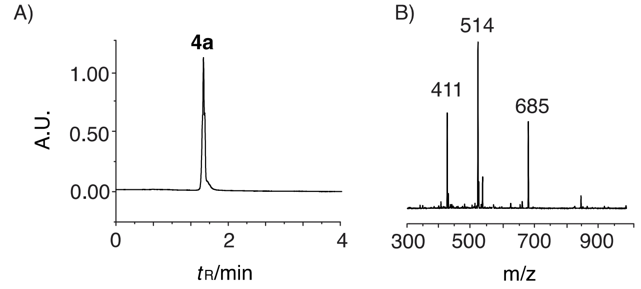

Figure S29. A) HPLC and B) ESI-MS analysis of purified compound **4a**

4b: Ac-c*agc*ag-βAla-$S(CH_2)_2SO_3H$; OD_{260} = 47.5; 52.6 nmol; 40 % yield; ε_{260} = 57800 L·mol^{-1}·cm^{-1}; t_R = 1.62 min; Formula: $C_{79}H_{108}N_{41}O_{21}S_2$, ESI-MS: $[M+5H]^{5+}$: 407.3 (calcd. = 407.4); $[M+4H]^{4+}$: 509.1 (calcd. = 509.0); $[M+3H]^{3+}$: 678.1 (calcd. = 678.4); MW_{dec} = 2032.87 (calcd. = 2032.09).

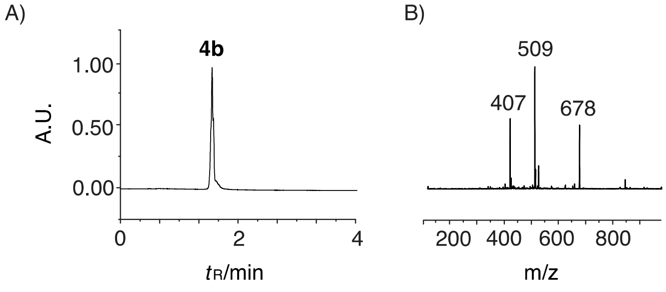

Figure S30. A) HPLC and B) ESI-MS analysis of purified compound **4b**.

5: Cys-cagcag-Lys; OD_{260} = 331; 258 nmol; 13 % yield; ε_{260} = 57800 L·mol^{-1}·cm^{-1}; t_R = 1.09 min; Formula: $C_{73}H_{98}N_{42}O_{18}S$, ESI-MS: $[M+5H]^{5+}$: 376.9 (calcd. = 377.8); $[M+4H]^{4+}$: 471.8 (calcd. = 471.9); $[M+3H]^{3+}$: 628.9 (calcd. = 628.9); $[M+2H]^{2+}$: 941.9 (calcd. = 942.9); MW_{dec} = 1883.88 (calcd. = 1883.89).

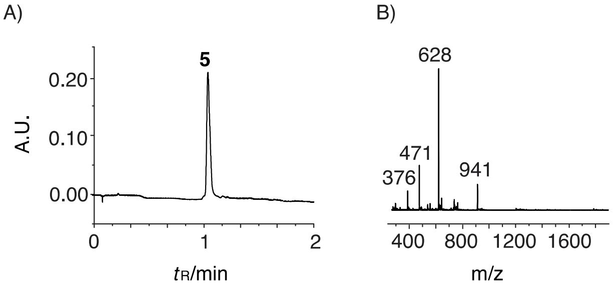

Figure S31. A) HPLC and B) ESI-MS analysis of purified compound **5**.

6: Ac-c*agc*ag-βAlaCys-cagcag-Lys; OD_{260} = 6.1; 460 pmol; 2 % yield; ε_{260} = 127600 $L\cdot mol^{-1}\cdot cm^{-1}$; t_R = 3.98 min; Formula: $C_{150}H_{201}N_{83}O_{36}S$, Maldi/Tof-MS: $[M+H]^{1+}$ = 3775.06 (calcd. = 3774.80).

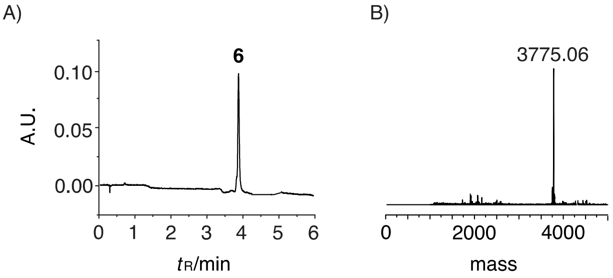

Figure S32. A) HPLC and B) Maldi/Tof-MS analysis of purified compound **6**.

17a: Ac-cagcagcag-βAla- $S(CH_2)_2CO$-Gly; OD_{260} = 29.8; 403 nmol; 30 % yield; ε_{260} = 95700 $L\cdot mol^{-1}\cdot cm^{-1}$; t_R = 0.87 min; Formula: $C_{106}H_{134}N_{60}O_{28}S$, ESI-MS: $[M+5H]^{5+}$: 546.8 (calcd. = 546.7); $[M+4H]^{4+}$: 683.2 (calcd. = 682.7); $[M+3H]^{3+}$: 910.1 (calcd. = 910.0); $[M+2H]^{2+}$: 1365.1 (calcd. = 1364.3); MW_{dec} = 2728.80 (calcd. = 2728.65).

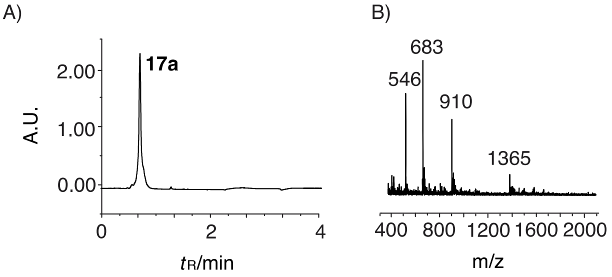

Figure S33. A) HPLC and B) ESI-MS analysis of purified compound **17a**.

17b: Ac-cagcagcag-βAla- $S(CH_2)_2SO_3H$; OD_{260} = 426; 45 nmol; 23 % yield; ε_{260} = 95700 $L\cdot mol^{-1}\cdot cm^{-1}$; t_R = 0.69 min; Formula: $C_{103}H_{129}N_{58}O_{29}S_2$, ESI-MS: $[M+4H]^{4+}$: 677.5 (calcd. = 677.5); $[M+3H]^{3+}$: 903.0 (calcd. = 903.0); $[M+2H]^{2+}$: 1354.1 (calcd. = 1353.9); MW_{dec} = 2708.39 (calcd. = 2707.62).

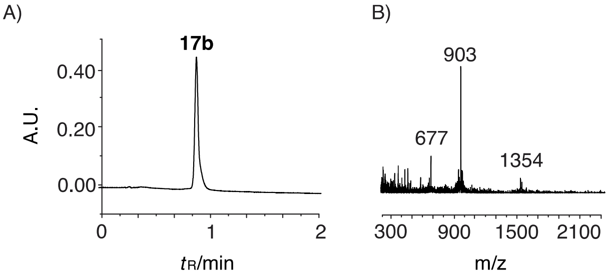

Figure S34. A) HPLC and B) ESI-MS analysis of purified compound **17b**.

18: Cys-cagcagcag-Gly; OD_{260} = 9.16; 115 nmol; 6 % yield; ε_{260} = 95700 $L \cdot mol^{-1} \cdot cm^{-1}$; t_R = 0.93 min; Formula: $C_{101}H_{128}N_{60}O_{26}S$, ESI-MS: $[M+5H]^{5+}$: 527.2 (calcd. = 527.1); $[M+4H]^{4+}$: 658.4 (calcd. = 658.5); $[M+3H]^{3+}$: 876.9 (calcd. = 877.8); $[M+2H]^{2+}$: 1315.8 (calcd. = 1316.2); MW_{dec} = 2630.63 (calcd. = 2630.54).

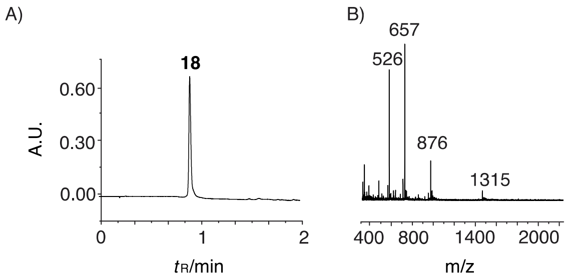

Figure S35. A) HPLC and B) ESI-MS analysis of purified compound **18**.

19: Ac-cagcagcag-βAlaCys-cagcagcag-Gly; OD_{260} = 1.15; 1.8 nmol; 64 % yield (peak area); ε_{260} = 191400 $L \cdot mol^{-1} \cdot cm^{-1}$; t_R = 2.97 min; Formula: $C_{202}H_{252}N_{118}O_{52}S$, ESI-MS: $[M+8H]^{8+}$: 650.3 (calcd. = 650.3); $[M+7H]^{7+}$: 743.5 (calcd. = 743.4); $[M+6H]^{6+}$: 867.0 (calcd. = 867.1); $[M+5H]^{5+}$: 1040.3 (calcd. = 1040.4); $[M+4H]^{4+}$: 1300.4 (calcd. = 1300.2); MW_{dec} = 5196.83 (calcd. = 5196.98).

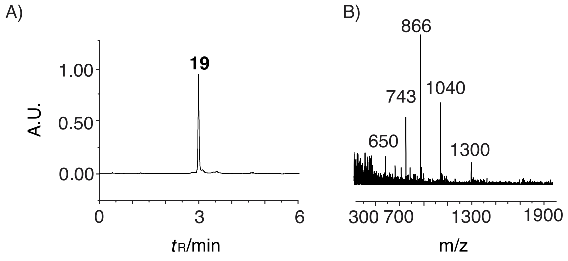

Figure S36. A) HPLC and B) ESI-MS analysis of purified compound **19**.

Measurements with PNA probes

Melting temperature (T_M) measurements

The PNA-DNA duplexes (1:1 stoichiometry) were dissolved in 1 µM concentration in 10 mM NaH_2PO_4 buffer pH 7.4 containing 150 mM NaCl. TCEP (20 mM) was added in case of cysteinyl-containing probes. Absorbance versus temperature profiles were obtained by monitoring at 260 nm by using a Varian Cary 100 UV spectrophotometer equipped with a peltier block, scanning from 95 °C to 20 °C with temperature increments of 0.5 °C per minute. The data (triplicates) were proceeded using OriginPro8 form OriginLab Corporation and T_M values derived from the maximum of the derivative curves.

Table S1. T_M values of PNA·DNA duplexes (R = $(CH_2)_2SO_3H$).

PNA	sequence	DNA	T_M / °C
1	Ac-cagcag-βAla-SR	5'-CTGCTG-3'	39
2	Cys-cagcag-Gly	5'-CTGCTG-3'	38
3	Ac-cagcag-βAla-Cys-cagcag-Gly	5'-CTGCTGCTGCTG-3'	56
17	Ac-cagcagcag-βAla-SR	5'-CTGCTGCTGCTG-3'	67
18	Cys-cagcagcag-Gly	5'-CTGCTGCTGCTG-3'	69
19	Ac-cagcagcag-βAla-Cys-cagcagcag-Gly	5'-CTGCTGCTGCTGCTGCTG-3'	74
4	Ac-c*agc*ag-βAla-SR	5'-CTGCTG-3'	40
5	Cys-cagcag-Lys	5'-CTGCTG-3'	45
6	Ac-c*agc*ag-βAla-Cys-cagc g-Lys	5'-CTGCTGCTGCTG-3'	59

Kinetic measurements of templated reactions

Reactive PNA probes were dissolved in 10 µM stock solution in ligation buffer (10 mM NaH_2PO_4, 150 mM NaCl, 10 mM MesNa, pH adjusted to 7.4). Mercaptopropionic thioester were incubated for 3 h with mercaptoethanesulfonate (10 mM) prior to use. The cysteinyl-PNA was incubated for 10 min in ligation buffer prior to use. For kinetic measurements appropriate amounts of PNA probes (final concentration 1 µM) and template (final concentration 200 nM or 100 nM) were mixed and incubated at 50 °C (or other *T* if mentioned different). The solution was agitated by means of a thermoshaker. Samples were withdrawn at certain time and quenched with 1.3 % TFA and subsequently analyzed by UPLC and Maldi-Tof/MS. In case partial blocker DNA was used the template was incubated with partial blocker DNA prior in 1:1 stoichiometry for 30 min prior to addition of reactive probes. The yield of ligation product was determined by means of HPLC analysis. For kinetic analysis data was submitted to nonlinear Michaelis-Menten fitting.

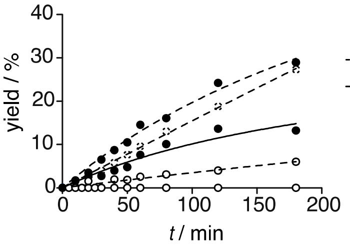

Figure S37. Time course of background ligation between reactive PNA probes **1** and **2** (dashed line) or between the aminomodified PNA probes **4** and **5** (full line) at different reaction temperatures.

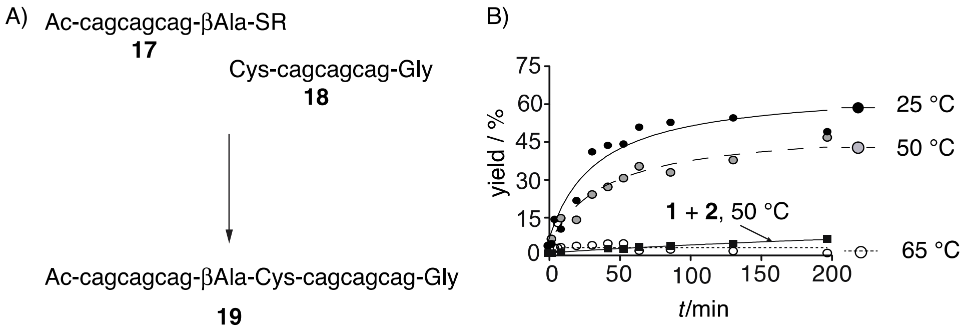

Figure S38. A) Native chemical ligation between size-expanded PNA probes **17** and **18**; B) Time course of background reaction in the absence of DNA template at different reaction temperatures,.

The background reaction rate is high, even at 50 °C. Increasing the reaction temperature to 65 °C results in reduced background but at this temperature the thioester hydrolyzes rapidly (data not shown) and is therefore not available for native chemical ligation.

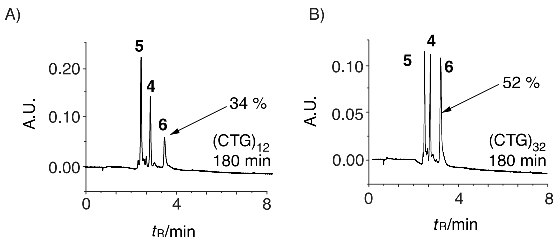

Figure S39. UPLC traces of native chemical PNA ligation reaction of aminoethyllysine modified PNA probes in presence of A) $(CTG)_{12}$ template or B) $(CTG)_{32}$ template.

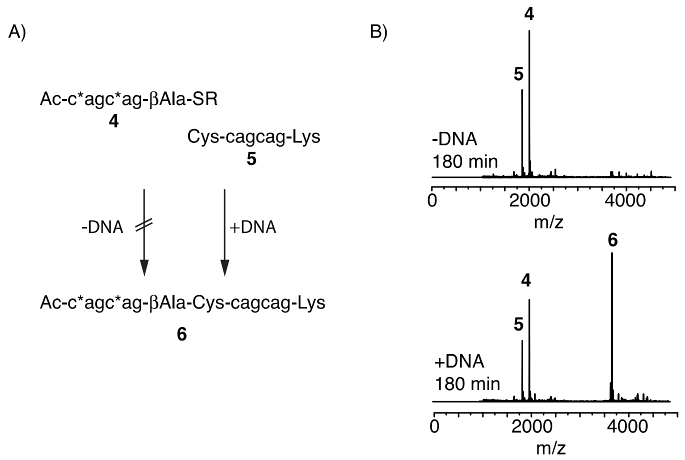

Figure S40. A) Native chemical ligation reaction between **4** and **5** in absence or in presence of DNA template ($(CTG)_{12}$); B) Maldi/Tof-MS analysis after 180 min reaction time.

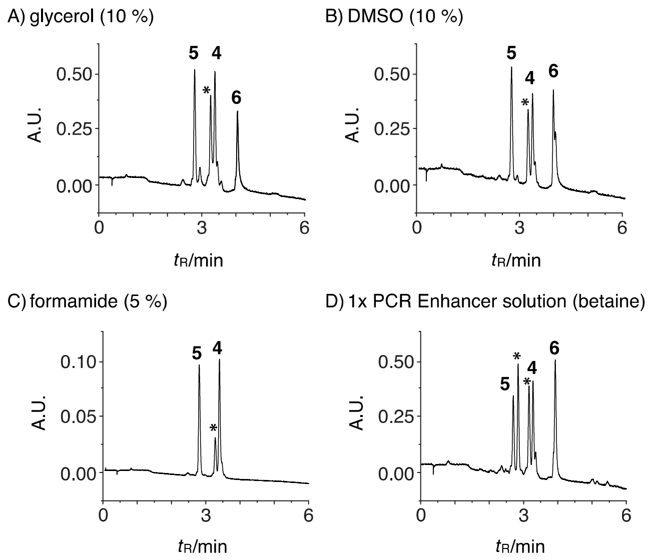

Figure S41. UPLC analysis of native chemical ligation between PNA **4** and **5** in presence of template $(CTG)_{32}$ and additives A) 10 % glycerol; B) 10 % DMSO; C) 5 % formamide; D) f PCR enhancer solution containing betaine; * markes the hydrolysis product of **4** or disulfide of **5**.

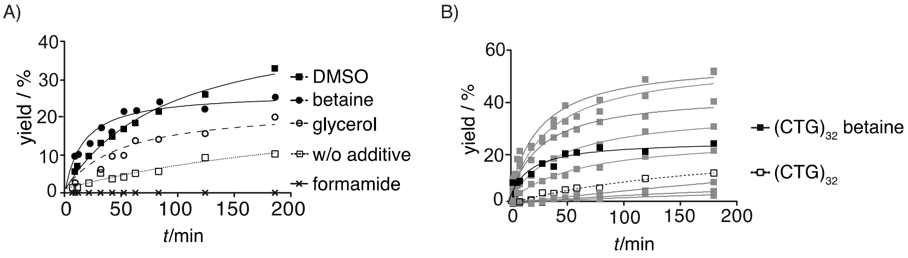

Figure S42. **A)** Time course **of native chemical ligation between** PNA **4** and **5 in presence of template** $(CTG)_{32}$ **and additives.** B) Comparison of reactions in buffers with or without betaine-.

Polymerase chain reaction (PCR) experiments

The PCR was performed with an iQ5 real-time PCR from BioRad. Primers were designed with Primer3 (www.bioinfo.ut.ee/primer3) to have annealing temperatures around 60 °C (Nearest Neighbor method). The sequences of primers used in this study are for the left primer, biotine-GACCCTGGAAAAGCTGATGA and for the right primer, Alexa700-GGCTGAGGAAGCTGAGGAG to amplify only across the CAG-repeat stretch of the IT15 gene. The biotin-tag is used for streptavidin-based isolation of the amplified double strand DNA and the fluorescence dye is used for quantification of amplified single strand target DNA. The genomic Huntington Disease DNA (HD-DNA) was purchased from Coriell Cell Repositories, USA and the genomic wild-type DNA (WT-DNA) was purchased from Roche. The Taq-polymerase Kit was purchased from peqlab containing reactionbuffer Y (10×), enhancer solution P, $MgCl_2$ and Taq-polymerase (5 uµL^{-1}). SYBRGold (10000×) was purchased by Invitrogen Molecular Probes and used in 10000-fold dilution. The dNTP, containing all 4 nucleobases was purchased as a premixed Kit by peqlab. Due to the high GC content of the IT15 dGTP and dCTP (both peqlab, 25 µM) were added additionally.

PCR conditions

The PCR reactions were performed in a volume of 100 µL mastermix containing (5 × 20.0 µL per well on a 96-well plate) DNA template (135 ng), reactionbuffer Y (10×, 12.0 µL), enhancer solution P (24.0 µL), $MgCl_2$ (25 mM, 6.0 µL), dNTP (10 mM, 2.40 µL), dCTP (5 mM, 0.96 µL), dGTP (5 mM, 0.96 µL), left primer (10 µM, 4.80 µL), right primer (10 µM, 4.80 µL), SYBRGold (6 µL) and Taq polymerase (1.20 µL). After an initial denaturation of 3 min at 95 °C 30 cycles of 10 sec at 95 °C, 20 sec at 60 °C and 20 sec at 72 °C were carried out followed by a cooling step at 4 °C.

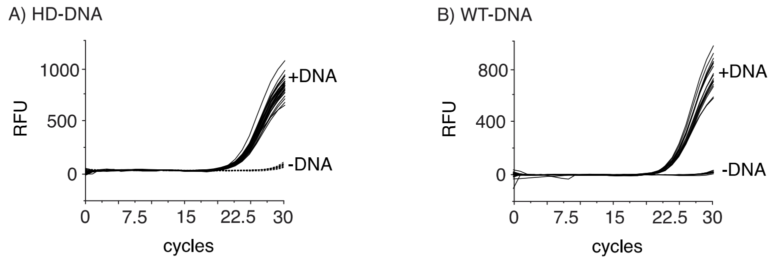

Figure S43. Real-time PCR amplification curves; A) PCR with HD-DNA and no template control (-DNA) and B) PCR with WT-DNA and no template control (-DNA).

Purification of dsDNA

The content of 40 wells was combined and the PCR product was separated from low molecular weight impurities by size exclusion with pre-packed spin columns from BioRad. The concentration of dsDNA was determined by means of Alexa Fluor 700 emission measured by using a NanoDrop ND-3300 Fluorospectrometer (excitation: 650 nm, emission: 723 nm). Typical yields of PCR reactions were 326 pmol (WT-DNA) and 269 pmol (HD-DNA).

Streptavidin binding procedure

Streptavidin-coated magnetic particles (20 mg, Roche) were washed twice with binding buffer (10 mM Tris-HCl, 1 mM EDTA, 100 mM NaCl, pH 7.5). dsDNA containing samples were diluted two-fold with binding buffer and added to the bead suspension. Suspensions were incubated for 2 h at room temperature with gently shaking. The beads were washed twice with washing buffer (10 mM Tris-HCl, 1 mM EDTA, 1 M NaCl, pH 7.5). Subsequently, a NaOH solution (100 µL, 0.1 M) was added and the resulting suspension was incubated for 5 min. The suspension was neutralized with NaOAc (25 µL, 3 M) and the released ssDNA precipitated in cold EtOH. The supernatant was removed after 15 min spin down and the precipitated ssDNA disolved in H_2O (30 µL). The ssDNA was quantified by fluorescence measurement of the Alexa Fluor 700 dye. Typical yields were 188 pmol for WT-DNA and 140 pmol for HD-DNA.

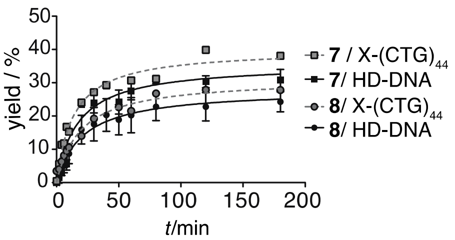

Figure S44. Kinetic measurements of HD-DNA (genomic and synthetic) in presence or absence of partial blocker DNA strand **7** or **8**.

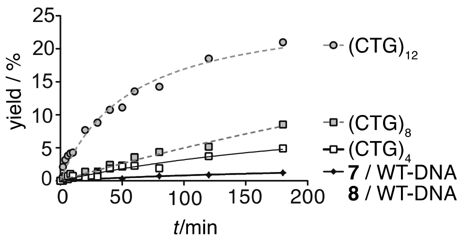

Figure S45. Kinetic measurements of WT-DNA (genomic and synthetic) in presence or absence of partial blocker DNA strand **7** or **8**.

Abbreviation

Boc	*tert*-butyloxycarbonyl
Cbz	benzyloxycarbonyl
DMF	*N*, *N*-dimethylformamide
DNA	deoxyribonucleic acid
dsDNA	double-stranded DNA
EDTA	ethylenediaminetetraacetic acid
Fmoc	9-fluorenylmethoxycarbonyl
HD	Huntington Disease
MBHA	*para*-methylbenzhydrylamide
MesNa	2-mercaptoethane sulfonate sodium
n.d.	not determined
NMM	*N*-methylmorpholine
PyBOP	benzotriazole-1-yl-oxy-tris-pyrrolidino-phosphonium hexafluorophosphate
ssDNA	single-stranded DNA
TFA	trifluoracetic acid
TFMSA	trifluormethanesulfonic acid
Tris	tris(hydroxymethyl)-aminomethan
Trt	trityl
WT	wild-type

References

1. G. Kallansrud and B. Ward, *Anal Biochem*, 1996, **236**, 134-138.
2. K. C. Engman, P. Sandin, S. Osborne, T. brown, P. Lincoln, B. Nordén, B. Albinsson and M. L. Wilhelmsson, *Nucl. Ac. Res.*, 2004, **32**, 5087-5095.
3. E. Uhlmann, A. Peyman, G. Breipohl and D. W. Will, *Angew. Chem. Int. Ed.*, 1998, **37**, 2796-2823.
4. S. Sforza, R. Corradini, S. Ghirardi, A. Dossena and R. Marchelli, *Eur J Org Chem*, 2000, 2905-2913.
5. G. Haaima, A. Lohse, O. Buchardt and P. E. Nielsen, *Angew. Chem.* , 2005, **108**, 2068-2070.
6. G. Kokotos, R. Verger and A. Chiou, *Chemistry European Journal*, 2000, **6**, 4211-4217.
7. I. Dilek, M. Madrid, R. Singh, C. D. Urrea and B. A. Armitage, *J Am Chem Soc*, 2005, **127**, 3339-3345.
8. G. Pattenden and T. Thompson, *Chem. Commun.*, 2001, 717-718.
